# Everolimus destabilizes thymidylate synthase via suppressing its O-GlcNAcylation and sensitizes HER2-negative breast cancer to fluorouracil

**DOI:** 10.1038/s41419-026-08715-z

**Published:** 2026-04-04

**Authors:** Xiao-Ting Jiang, Huipei Gan, Shaoyi Wang, Linghan Wang, Zijie Cai, Lok Lam Wong, Jingru Wang, Mengdi Zhu, Nianqiu Liu, Wang Yang, Yujie Liu, Liang Jin, Yudong Li, Shunying Li, Qianfeng Shi, Jinna Lin, Keyang Qian, Qi Wang, Xin Yang, Zhuangqiu Yang, Yingyan Chen, Yinxia Wu, Qiang Liu

**Affiliations:** 1 Guangdong Provincial Key Laboratory of Malignant Tumor Epigenetics and Gene Regulation, Guangdong-Hong Kong Joint Laboratory for RNA Medicine, Sun Yat-sen Memorial Hospital, Sun Yat-sen University, Guangzhou, China; 2 Breast Tumor Center, Sun Yat-sen Memorial Hospital, Sun Yat-sen University, Guangzhou, China

**Keywords:** Breast cancer, Targeted therapies

## Abstract

5-Fluorouracil (5-FU) and its prodrugs are widely used drugs for chemotherapy in various cancers. However, their effectiveness in breast cancer is limited. mTORC1 pathway, as a known mediator of therapy resistance in breast cancer, presents a compelling target for combination approaches. This study investigated the mTORC1 inhibitor everolimus as a sensitizer to 5-FU and capecitabine in breast cancer, exploring thymidylate synthase (the direct target of 5-FU) as a predictive biomarker and targeted mechanism. Our results demonstrate that everolimus significantly enhances 5-FU efficacy in HER2-negative breast cancer in vivo and in vitro. Mechanistically, everolimus downregulates thymidylate synthase (TYMS) by inducing its proteasomal degradation through a ubiquitination-independent way involving downregulation of O-GlcNAc transferase (OGT) and a reduced O-GlcNAcylation of TYMS, which destabilizes TYMS homodimer. Overexpression of OGT reversed TYMS degradation. Importantly, this combination strategy was effective in refractory breast cancer patients, and decreased levels of TYMS and OGT were observed in breast cancer patient specimens collected before and after everolimus-containing treatment. In conclusion, our study reveals that everolimus sensitizes breast cancer to fluoropyrimidines by destabilizing TYMS through modulation of its O-GlcNAcylation. These findings support a promising combination strategy to improve the therapeutic efficacy of 5-FU and capecitabine in breast cancer.

## Introduction

For decades, 5-Fluorouracil (5-FU) and its oral prodrugs (e.g., capecitabine, tegafur) have been cornerstone chemotherapy drugs for various cancers, including breast cancer and gastrointestinal cancer. Since the 1970s, 5-FU has maintained an important role in adjuvant breast cancer regimens, and the phase III CREATE-X/JBCRG-04 trial further demonstrated that capecitabine significantly prolonged overall survival in patients with HER2-negative breast cancer [1]. These antimetabolites exert cytotoxicity by substituting dUMP with fluorodeoxyuridine monophosphate (FdUMP), thereby inhibiting thymidylate synthase (TYMS), a critical enzyme in DNA synthesis [2, 3]. Despite targeting this essential replicative process, the efficacy of 5-FU in breast cancer is limited, as evidenced by the GIM2 study showing the addition of fluorouracil failed to optimize epirubicin, cyclophosphamide, and then paclitaxel (EC-P) based adjuvant chemotherapy for patients with high-risk early-stage disease [4, 5]. Therefore, improving the clinical utility of 5-FU remains a critical challenge.

The cytotoxic effects of chemotherapeutic agents, including 5-fluorouracil (5-FU), activate stress-responsive pathways in cancer cells. Although this activation can initiate cell death, it also facilitates cellular adaptation, leading to treatment refractory. The mTORC1 pathway, a stress-responsive signaling hub, is a central driver of tumor growth and a known mechanism of resistance to diverse therapies [6–10]. In the BOLERO-1, -2, -3 trials, adding everolimus (an FDA-approved mTORC1 inhibitor) to chemotherapy and HER2-targeted therapy, or endocrine therapy significantly improved the treatment response in advanced breast cancer [11–13]. Furthermore, mTOR inhibition can override resistance to other chemotherapeutics, such as topoisomerase II inhibitors [14]. A clinical trial in HER2-negative metastatic breast cancers reported that the combined use of everolimus and capecitabine achieved good efficacy with 50% clinical benefit rate (CBR) and median progression-free survival (PFS) more than 6 months [15], suggesting that everolimus may synergize with fluorouracil in breast cancer treatment.

In this study, we found that mTORC1 inhibition by everolimus significantly sensitized several HER2-negative breast cancer cell lines to fluorouracil both in vitro and in vivo. Further mechanistic study demonstrated that everolimus downregulated TYMS, a direct target of fluorouracil, by modulating its protein stability. In addition, we found that everolimus downregulated OGT to decrease the O-GlcNAcylation of TYMS, which led to its dissociation into monomers and ubiquitin-independent degradation.

## Results

### Everolimus increases HER2-negative breast cancer susceptibility to fluoropyrimidine

**Fig. 1:**
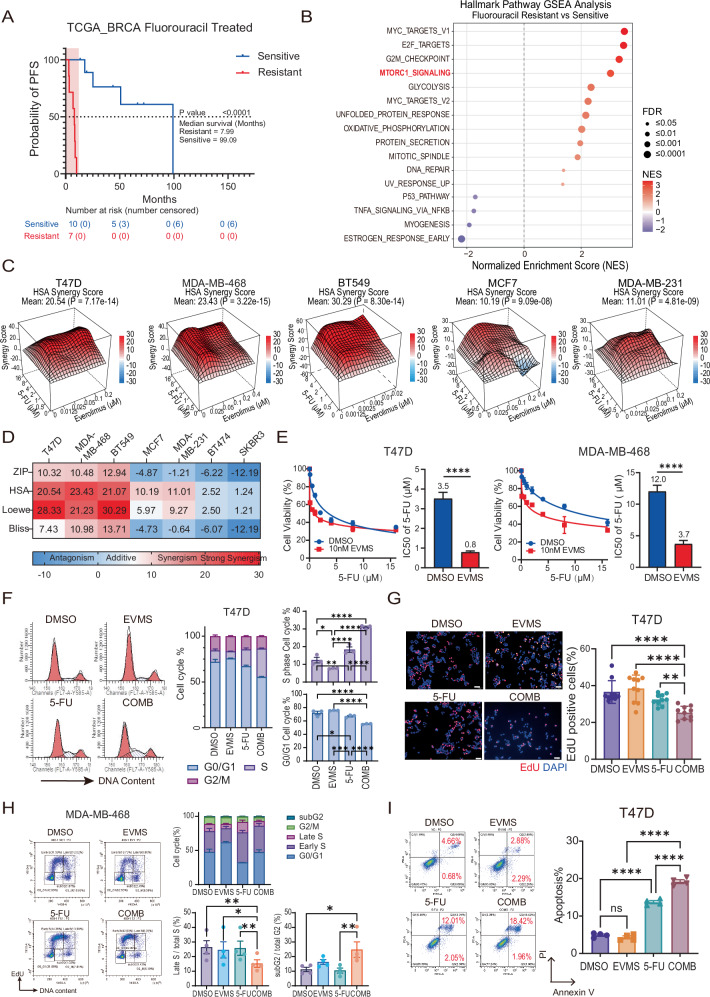
Everolimus increases HER2-negative breast cancer susceptibility to fluoropyrimidine. **A** Kaplan–Meier survival analysis of fluorouracil or capecitabine-treated breast cancer patients (*n* = 17) from the TCGA PanCancer Atlas (2018). Progression-free survival (PFS) following fluorouracil or capecitabine-containing treatment was applied to stratify Sensitive or Resistant patients. Shorter than 12 months were considered resistant, otherwise were sensitive. Statistical significance was determined by Log-rank (Mantel–Cox) test. **B** Significant (FDR < 0.05) GSEA enrichment plots for Hallmark pathways comparing Resistant and Sensitive patients from (**A**). **C** Heatmaps of synergistic effects of everolimus (EVMS) and 5-fluorouracil (5-FU) combination treatment in different breast cancer cell lines (ER-positive: MCF7, T47D; Triple-negative: MDA-MB-468, BT549, MDA-MB-231). Cells were treated for 4 days. Data represents meaning from at least three biological replicates. **D** Mean synergy score was calculated using ZIP, Loewe, HSA, and Bliss models, with values > 20 indicating strong synergism, >10 synergy, 0–10 additive effects, and values < 0 antagonism. **E** Dose–response curve and IC50 of 5-FU in T47D and MDA-MB-468 cells under vehicle (DMSO) or 10 nM everolimus (EVMS) treatment. Cells were treated for 96 h, and cell viability was measured by MTT assay and normalized to untreated cells. Each data point represents at least three replicates. The dose–response curves were fitted using a nonlinear regression model, and the IC50 values are presented as mean ± SEM and compared as unshared parameters by the extra sum-of-squares F test. **F** Cell cycle analysis in T47D cells treated with vehicle (DMSO), 10 nM everolimus, 0.5 µM 5-FU, or their combination for 48 h. Bar graphs show the proportion of cells in each cell cycle phase (middle) and detailed proportions for S phase and G0/G1 phase (right). Individual dots represent biological replicates. Data are shown as mean ± SEM from three independent replicates, and statistical significance was determined by two-way ANOVA. **G** EdU incorporation assay in T47D cells treated with vehicle (DMSO), 10 nM everolimus, 0.5 µM 5-FU, or their combination for 48 h. Bar graphs depict the percentage of EdU-positive cells. Data are mean ± SEM from ten random fields per condition across three biological replicates, and statistical significance was determined by one-way ANOVA. The scale bar represents 100 µm. **H** EdU-based cell cycle analysis in MDA-MB-468 cells treated with vehicle (DMSO), 10 nM everolimus, 0.5 µM 5-FU, or their combination for 48 h. Bar graphs show the proportion of cells in each cell cycle phase (top right) and the detailed proportion of late S phase (within total S phase) and subG2 phase (within total G2/M phase) (bottom right). Data are shown as mean ± SEM from four independent replicates, and statistical significance was determined by one-way ANOVA. **I** Apoptosis assay in T47D cells treated with vehicle (DMSO), 10 nM everolimus, 0.5 µM 5-FU, or the combination for 72 h. The bar graph depicts the proportion of Annexin V-positive cells. Data represent mean ± SEM from four biological replicates, with statistical analysis performed by one-way ANOVA. Ns, not significant; *P < 0.05; **P < 0.01; ***P < 0.001; ****P < 0.0001.

To investigate the role of the mTORC1 pathway in fluoropyrimidine sensitivity, we analyzed RNA sequencing data from 17 breast cancer patients treated with fluorouracil or capecitabine from the TCGA database [16]. Patients were classified as resistant (*n* = 7) if disease progressed within 12 months (PFS < 12 months), or sensitive (*n* = 10) if no progress or recurrence occurred within this period (Fig. 1A), and two groups showed no significant differences in age at diagnosis or AJCC TNM stage (Fig. S1A, B). Hallmark pathways Gene Sets Enrichment Analysis (GSEA) revealed significant enrichment in mTORC1_signalling pathway (FDR < 0.0001) and the other oncogenic pathways in resistant group (Fig. 1B), suggesting these pathways drive aggressive tumor behavior and contribute to shorter progression-free survival after fluoropyrimidine treatment.

Given the enrichment of mTORC1 signaling in resistant patients, we further evaluated the combination efficacy of 5-FU and everolimus in breast cancer cell lines, including ER-positive (MCF7, T47D), HER2-positive (BT474, SKBR3), and triple-negative (MDA-MB-468, BT549, and MDA-MB-231) subtypes. This combination exhibited universal synergy (HSA Synergy Score >10) in ER-positive and triple-negative subtypes but not HER2-positive cell lines (Figs. 1C and S1C–E), while the degree of synergism varied, with T47D, MDA-MB-468 and BT549 showing stronger synergism (HSA Synergy Score >20) than MCF7 and MDA-MB-231 (HSA Synergy Score 10-20) (Fig. 1D), underscoring the synergy observed in HER2-negative breast cancer and highlighting the critical need to define predictive biomarkers. Additionally, we assessed the IC50 of 5-FU with or without everolimus co-treatment in T47D, MDA-MB-468 and BT549 models (Figs. 1E and S1F) and the continuous exposure to a clinically relevant dose of everolimus (10 nM) markedly reduced the IC50 of 5-FU by 77% in T47D and 69% in MDA-MB-468 cells (Fig. 1E). Notably, even 1 nM everolimus sensitized BT549 cells to 5-FU, reducing its IC50 by 68% (Fig. S1F).

The antimetabolic activity of fluoropyrimidine disrupts DNA synthesis by substituting normal deoxynucleotides, leading to replicative stress in the S phase, chromatin instability, and apoptosis. While everolimus monotherapy, as an mTORC1 inhibitor, reduced the S-phase population in T47D cells (*P* = 0.0406) [17], 5-FU alone increased it (*P* = 0.0071) (Fig. 1F). In both T47D and BT549 cell lines, the combination treatment led to further accumulation in the S phase compared to 5-FU alone (T47D, *P* < 0.0001; BT549, *P* < 0.0001) (Figs. 1F and S1G), while EdU incorporation declined following combination therapy (COMB vs 5-FU in T47D, *P* = 0.0047; BT549, *P* = 0.0005) (Figs. 1G and S1H). Non-EdU-incorporated S-phase cells endure replication forks that stall or collapse [18], suggesting compromised DNA synthesis, which leads to DNA double-strand breaks and apoptosis. Applying co-staining of EdU and DNA content provided detailed DNA synthesis profiling during cell cycle program in MDA-MB-468 cells, which directly validates replication stalling. Combination treatment significantly decreased the population of EdU-positive cells in late S phase (with near-4N DNA content) (to DMSO, *P* = 0.005; to EVMS, *P* = 0.0149; to 5-FU, *P* = 0.0074) while increasing the sub-G2 population (EdU-negative with DNA content lower than G2/M) (to DMSO, *P* = 0.0126; to 5-FU, *P* = 0.0094) (Fig. 1H). This pattern is characteristic of replication stress during elongation.

We also found that combination treatment decreased EdU incorporation in MCF7 cells and MDA-MB-231 cell lines when compared to vehicle (DMSO) or single agent (COMB vs 5-FU in MCF7, *P* = 0.019; MDA-MB-231, *P* < 0.0001) (Fig. S2A), consistent with the synergism effects found in these two cell lines. Besides, adding everolimus to 5-FU treatment significantly reduced colony formation (MCF7, *P* = 0.0043; T47D, *P* = 0.042; MDA-MB-468, *P* = 0.0017) (Fig. S2B) and increased Annexin V-positive apoptotic cells (T47D, *P* < 0.0001; MCF7, *P* = 0.0048; BT549, *P* < 0.0001) (Figs. 1I and S2C) in different ER-positive and triple-negative breast cancer cell lines.

In conclusion, everolimus enhances the efficacy of fluoropyrimidines in HER2-negative breast cancer cell lines by sensitizing cancer cells to chemotherapy-induced replication stress, ultimately disrupting proliferation and promoting apoptosis. The observed variability in synergistic response highlights the necessity for identifying predictive biomarkers to guide patient selection for this combination therapy.

### Thymidylate synthase is a prognostic biomarker and predictive target for combination therapy in HER2-negative breast cancer

**Fig. 2:**
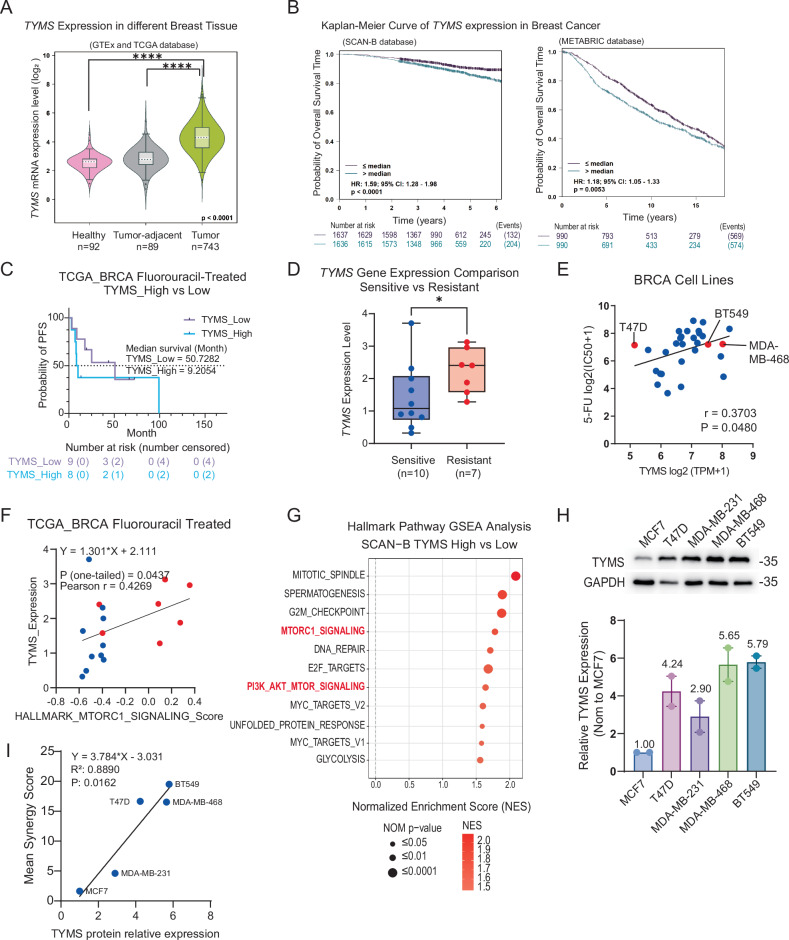
Thymidylate synthase is a prognostic biomarker and predictive target for combination therapy in HER2-negative breast cancer. **A** Violin plot of *TYMS* expression in healthy breast, tumor-adjacent, and breast cancer tissues. Data was obtained from the GTEx and TCGA databases. **B** Kaplan–Meier survival curves for overall survival analysis of breast cancer patients stratified by *TYMS* expression levels from the SCAN-B and METABRIC databases. The cutoff was set at the median value. Hazard ratios (HR) with 95% confidence intervals and log-rank P values are shown. **C** Kaplan–Meier survival analysis of TYMS_high and TYMS_low samples from Fig. 1A. Two groups were divided by the median *TYMS* expression level. Progression-free survival (PFS) following fluorouracil or capecitabine-containing treatment was compared between two groups, demonstrating a shorter median PFS time in the TYMS high group. **D** Comparison of *TYMS* expression level of Resistant and Sensitive patients from Fig. 1A. Statistical significance was assessed by one-tailed unpaired *t* test with Welch’s correction (*P = 0.0363). **E** Pearson correlation analysis between TYMS expression (log2(TPM + 1)) and IC50 of fluorouracil (log2(IC50 + 1)) in breast cancer cell lines. Gene expression data were from CCLE, and IC50 data from GDSC. Pearson correlation coefficient (*r*) and *P* values are shown. **F** Pearson correlation analysis between the ssGSEA score of Hallmark_mTORC1_signalling pathway from patients from Fig. 1A with their TYMS expression. Blue dots were sensitive samples, and red dots were resistant samples. The equation of simple linear regression, Pearson correlation coefficient and *P* values are shown. **G** Significant (Nominal *P* < 0.05) GSEA enrichment plots for Hallmark pathways comparing TYMS-high and low breast cancer patients from the SCAN-B database. **H** Western blot of TYMS protein in different breast cancer cell lines. Relative TYMS protein expression in breast cancer cell lines normalized to the housekeeping gene (GAPDH) and further normalized to MCF7. Data from two independent experiments are shown as mean ± SEM. **I** Pearson correlation analysis between TYMS protein expression from (**H**) and mean synergy score from Fig. 1D across five breast cancer cell lines (T47D, MCF7, MDA-MB-231, MDA-MB-468, and BT549). The equation of simple linear regression, Pearson correlation coefficient (*r*) and *P* values are shown.

To identify biomarkers predictive of the observed synergy, we investigated thymidylate synthase (TYMS), the direct target of 5-FU. TYMS expression is a well-established determinant of fluoropyrimidine resistance, where its downregulation sensitizes cells to treatment [19–24]. Consistent with its role as a therapeutic target, TYMS expression was significantly elevated in breast tumors compared to both healthy and adjacent normal tissues in GTEx and TCGA datasets (*P* < 0.0001) (Fig. 2A). Clinically, high TYMS expression correlated with poorer prognosis across independent cohorts, including SCAN-B [25] (HR = 1.59, *P* < 0.0001), METABRIC [26] (HR = 1.18, *P* = 0.0053) and Affymetrix [27] (HR = 1.63, *P* < 0.0001) databases (Figs. 2B and S3A). At the subtype level, TYMS expression was highest in basal-like and luminal B cancers (Fig. S3B). Notably, elevated TYMS was associated with worse outcomes specifically in basal-like (HR = 2.2, *P* = 0.0068) and luminal (HR = 1.6, *P* = 0.039) subtypes, but not in the HER2-enriched subtype (Fig. S3C). These findings validate TYMS as a prognostic biomarker and underscore its relevance in HER2-negative breast cancers, aligning with the therapeutic context of our combination strategy.

Consistent with its mechanistic role, *TYMS* expression was predictive of treatment response. In the TCGA fluorouracil-treated cohort, patients with high *TYMS* levels (stratified by median) exhibited a shorter median survival (9 vs. 50 months; Fig. 2C). Resistant patients (PFS < 12 months) also exhibited significantly higher *TYMS* expression (*P* = 0.0363) (Fig. 2D). Supporting this, analysis of Cancer Cell Line Encyclopedia (CCLE) and Genomics of Drug Sensitivity in Cancer (GDSC) data revealed a positive correlation between *TYMS* expression and the IC50 of 5-FU (*P* = 0.048), confirming that higher TYMS confers intrinsic 5-FU resistance in breast cancer models (Fig. 2E).

Given the enrichment of mTORC1 signaling in fluoropyrimidine-resistant patients (Fig. 1B), we interrogated the relationship between TYMS and this pathway. We found a positive correlation between *TYMS* expression and the Hallmark mTORC1 signaling pathway single-sample GSEA (ssGSEA) score in fluorouracil-treated TCGA_BRCA cohort (*P* = 0.0437) (Figs. 2F and S3D). This link was corroborated in the SCAN-B database, where high *TYMS* expression was associated with enrichment of both mTORC1 signaling and PI3K/AKT/mTOR signaling pathways (Figs. 2G and S3E). This positions TYMS as a candidate biomarker for identifying tumors likely to benefit from combined mTORC1 and 5-FU inhibition.

To validate this, we assessed TYMS protein levels across a panel of HER2-negative breast cancer cell lines (Fig. 2H). Strikingly, we observed a significant positive correlation between basal TYMS protein expression and the synergy score of 5-FU and everolimus combination therapy (*P* = 0.0162; Fig. 2I).

In summary, these data establish TYMS as an integrated biomarker that correlates with poor prognosis, intrinsic 5-FU resistance, and mTORC1 pathway activation, and directly predicts the degree of therapeutic synergy achieved by combining 5-FU with everolimus.

### Everolimus enhances fluorouracil sensitivity by downregulating thymidylate synthase

**Fig. 3:**
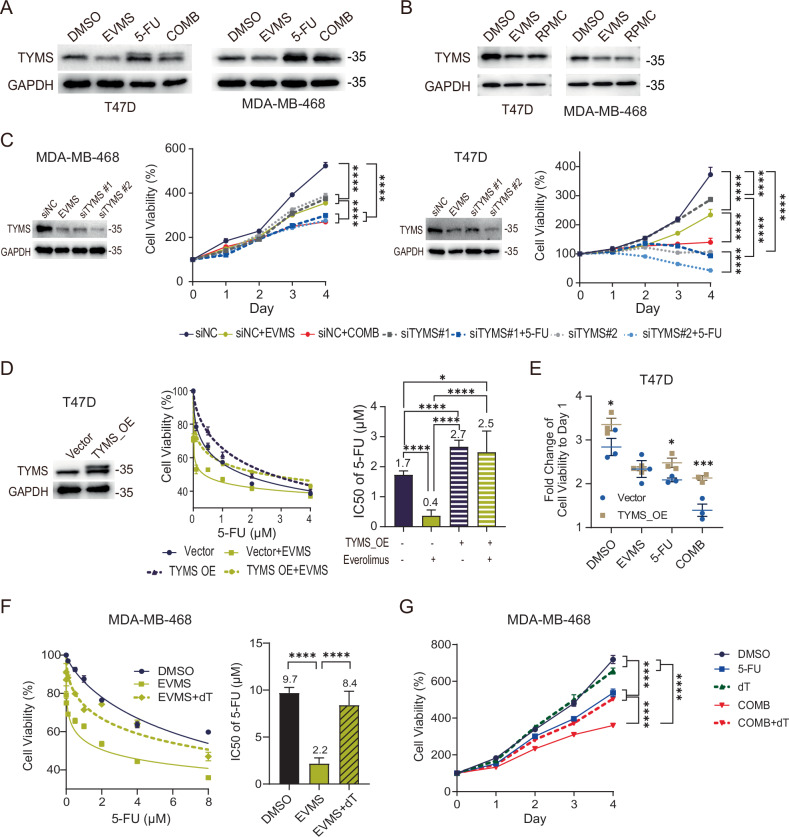
Everolimus enhances fluorouracil sensitivity by downregulating thymidylate synthase. **A** Western blot analysis of the indicated cell lines under vehicle (DMSO), 10 nM everolimus (EVMS), 0.5 µM 5-fluorouracil (5-FU), or EVMS + 5-FU (COMB) for 48 h. **B** Western blot analysis of the indicated cell lines under vehicle (DMSO), 10 nM everolimus (EVMS), or 10 nM rapamycin (RPMC) for 24 h. **C** MDA-MB-468 and T47D cells were transfected with either a negative control siRNA (siNC) or TYMS siRNAs (siTYMS #1, siTYMS #2) for 48 h, followed by treatment with 10 nM EVMS and/or 0.5 µM 5-FU as indicated. TYMS silencing was validated by western blot (with 48-h EVMS treatment serving as a positive control). Cell viability was measured by MTT assay at different time points. Data from three independent experiments are shown as mean ± SEM, and statistical significance was assessed by two-way ANOVA. **D** T47D cells were transfected with a control (Vector) or TYMS overexpression (TYMS_OE) plasmid. Left panel: Validation of TYMS overexpression by western blot. Right panel: Dose–response curves and IC50 values for 5-FU, with or without 10 nM everolimus (EVMS), measured after 96 h. Data shown as mean ± SEM from three independent experiments. Curves were fitted using a nonlinear regression model and statistically compared using an extra sum-of-squares F test. **E** Fold change in cell viability from Day 1 to Day 4, based on growth curves of T47D_NC or TYMS_OE cell lines treated with DMSO, 10 nM EVMS, 0.5 µM 5-FU, or the combination (COMB). Results from three independent experiments are presented as mean ± SEM, and statistical significance was determined by two-way ANOVA. **F** Dose–response curve and IC50 of 5-FU in MDA-MB-468 cells treated with or without 10 nM everolimus (EVMS) or 1 µM thymidine (dT) for 96 h. Data represents three independent experiments shown as mean ± SEM and analyzed by extra sum-of-squares F test. **G** Growth curve of MDA-MB-468 cells treated with DMSO, 0.5 µM 5-FU, 1 µM thymidine (dT), 10 nM everolimus + 0.5 µM 5-FU (COMB), and COMB + 1 µM dT. Results from three independent experiments are shown as mean ± SEM and were analyzed by two-way ANOVA. *P < 0.05; ***P < 0.001; ****P < 0.0001.

To resolve the paradox whereby high TYMS expression confers 5-FU resistance yet predicts enhanced sensitivity to everolimus combination therapy, we examined the effect of mTORC1 inhibition on TYMS. As previously reported, 5-FU treatment increased TYMS protein levels, a phenomenon attributed to metabolite FdUMP-mediated protein stabilization that contributes to resistance [28–30] (Fig. 3A). Everolimus alone significantly decreased TYMS protein and partially reversed the 5-FU-induced stabilization (Fig. 3A). We confirmed that rapamycin, another mTOR inhibitor, also reduced TYMS protein, indicating an on-target class effect (Fig. 3B). Everolimus-induced TYMS suppression was time-dependent but not dose-dependent within the 10–100 nM clinical range (Fig. S4A, B), indicating that standard FDA-approved doses can directly target TYMS.

To determine the specificity for TYMS, we examined other key enzymes in fluorouracil metabolism: thymidine phosphorylase (TYMP), uridine monophosphate synthetase (UMPS), dihydropyrimidine dehydrogenase (DPYD), thymidylate synthase (TYMS), and thymidine kinase 1 (TK1). TYMS was the only protein consistently downregulated by everolimus across all four cell lines tested, without mRNA expression change (Fig. S4C, D), underscoring its central role and suggesting post-transcriptional regulation.

Functional validation using TYMS knockdown in MDA-MB-468 and T47D cells reduced cell viability similarly to everolimus alone. Subsequent 5-FU exposure further decreased viability (*P* < 0.0001), mirroring the combination treatment in control cells (Fig. 3C). Conversely, TYMS overexpression in T47D cells conferred resistance to 5-FU and abolished everolimus-induced sensitization (Fig. 3D). The proliferation of TYMS-overexpressing cells under combination treatment exceeded that of control cells, approaching levels seen with 5-FU alone (Figs. 3E and S4E).

As thymidine kinase 1 (TK1) provides an alternative dTMP source via the salvage pathway, bypassing TYMS-mediated de novo synthesis, we supplemented MDA-MB-468 cells with thymidine (dT). This supplementation rescued the everolimus-induced sensitization to 5-FU (Fig. 3F), and growth activity under combination treatment increased significantly with thymidine addition (Fig. 3G). These findings strongly support TYMS as the primary mediator of everolimus-induced fluorouracil sensitization.

In conclusion, though high TYMS expression is a poor prognostic factor in HER2-negative breast cancer and correlates with intrinsic 5-FU resistance, everolimus directly counteracts this resistance by downregulating TYMS protein, thereby sensitizing tumors to fluoropyrimidine therapy. These findings establish TYMS as the central therapeutic target for this rational drug combination.

### Everolimus promotes ubiquitin-independent proteasomal degradation of TYMS

**Fig. 4:**
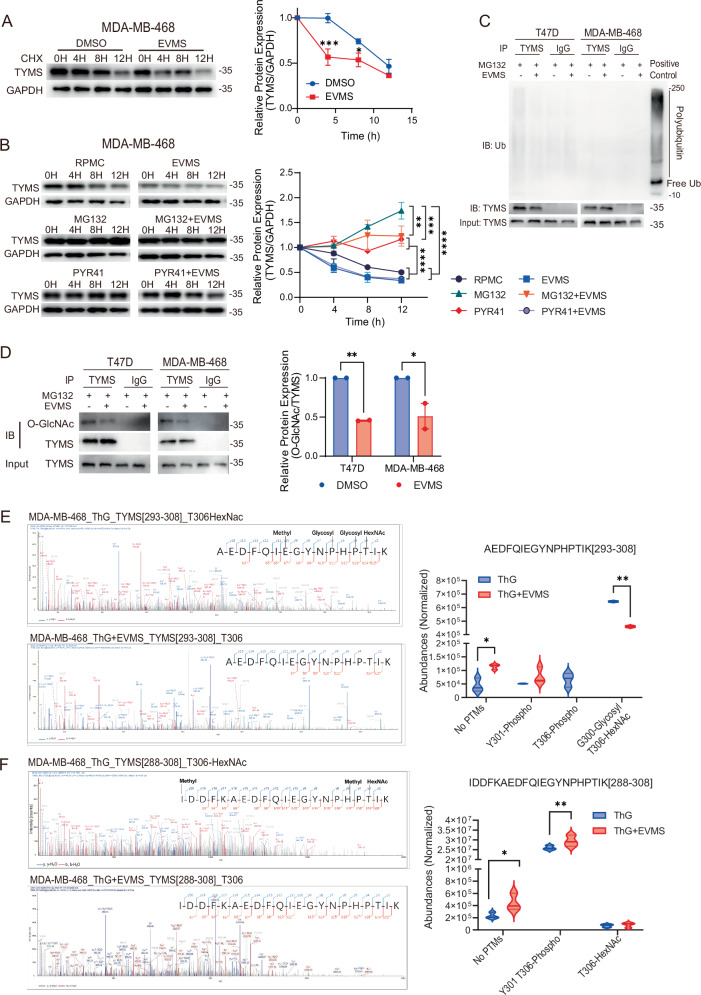
mTORC1 inhibition destabilizes thymidylate synthase via O-GlcNAcylation. **A** Treatment with 10 nM everolimus induced faster degradation of TYMS protein in MDA-MB-468 cell lines. Cells were treated with 100 µg/ml cycloheximide (CHX) and either vehicle (DMSO) or 10 nM everolimus (EVMS) for the indicated time points. Data from two independent experiments are shown as mean ± SEM, and significance was determined using two-way ANOVA. **B** Western blot analysis and quantification of MDA-MB-468 cells treated with 100 nM rapamycin (RPMC), 100 nM everolimus (EVMS), 5 µM MG132, 5 µM PYR-41, or their combinations for the indicated durations. Densitometry was normalized to GAPDH and the 0-h time point. Data from two independent experiments are shown as mean ± SEM, and statistical significance was determined by two-way ANOVA. **C** Ubiquitination assay of T47D and MDA-MB-468 cells treated with 5 µM MG132, with or without 100 nM everolimus, for 12 h. 2.5% input was used as a loading control, and whole-cell lysate served as the positive control. **D** O‑GlcNAcylated TYMS was detected by co-immunoprecipitation using an anti‑thymidylate synthase (TYMS) antibody or an IgG control in T47D and MDA-MB-468 cells treated with MG132 (5 µM) with or without everolimus (100 nM) for 12 h. The immunoprecipitated proteins were blotted with an O‑GlcNAc antibody. 2.5% of the whole lysate was used as input, and the relative levels of O‑GlcNAcylated TYMS were normalized to the total pulldown TYMS. The bar graph shows the mean ± SEM of the relative O‑GlcNAcylated TYMS levels from two independent experiments, and statistical significance was determined by multiple *t* tests. **E**, **F** TYMS is O-GlcNAcylated (HexNAc) at T306. MDA-MB-468 cells were treated with Thiamet-G (2 µM; Th-G, an OGA inhibitor) with or without everolimus (100 nM) for 12 h. The HCD-MS/MS spectrum of peptides spanning residues **E** 293–308 and **F** 288–308, which covers the T306 O-GlcNAcylated (HexNAc) site of TYMS, is shown. The modified residue is highlighted in the peptide sequence. Left panels were representative figures, and the right panels are the violin plot of label‑free quantitation of post‑translationally modified TYMS peptides containing the T306 amino acid. Data are expressed as normalized abundance and are presented as violin plots from three replicates. Statistical significance was determined by multiple *t* tests. *P < 0.05; **P < 0.01; ***P < 0.001; ****P < 0.0001.

To elucidate the mechanism underlying mTORC1 inhibitor-induced TYMS protein depletion, we used cycloheximide (CHX) to suppress overall protein synthesis, allowing us to determine whether TYMS reduction resulted from inhibited synthesis or enhanced degradation. TYMS exhibited a slow synthesis rate, showing no significant increase over 12 h with or without everolimus (Fig. S4F), indicating the drug does not inhibit its synthesis. Instead, everolimus accelerated TYMS degradation after CHX exposure in both MDA-MB-468 and T47D cells (Figs. 4A and S4G), indicating reduced protein stability.

Using pathway-specific inhibitors, we found that only the proteasome inhibitor MG132 reversed everolimus-induced TYMS depletion, while autophagy inhibitors (3-Methyladenine (3-MA), Bafilomycin A1 (Baf A1), hydroxychloroquine (HCQ)) had no effect (Fig. S4H). suggesting degradation occurs via the ubiquitin-proteasome system (UPS).

Tracking TYMS level in MG132-treated MDA-MB-468 cells showed a gradual increase and rescued the everolimus-induced depletion (Fig. 4B). Although proteasomal degradation typically occurs via ubiquitin-dependent mechanisms, some proteins, including TYMS, undergo ubiquitin-independent degradation [31]. Since MG132 covalently binds to the proteasome core, the final effector, it indiscriminately inhibits both pathways. To distinguish between them, we used PYR-41, an inhibitor of ubiquitin-dependent degradation targeting the E1 activating enzyme. PYR-41 failed to restore TYMS levels in MDA-MB-468 cells (Fig. 4B), and T47D cells (Fig. S4I), where MG132 was effective. Furthermore, immunoprecipitation assays detected no ubiquitin labeling on TYMS under any condition (Fig. 4C). MG132 was included to allow the accumulation of post-translational modification of target proteins before degradation, such as polyubiquitination, which are otherwise rapidly degraded and difficult to detect.

These results confirm that everolimus enhances the ubiquitin-independent proteasomal degradation of TYMS, thereby sensitizing breast cancer cells to fluoropyrimidines.

### Decreased O-GlcNAcylation destabilizes thymidylate synthase dimer in breast cancer cells

In the classical proteasomal degradation pathway, the regulatory particle of the proteasome initiates protein degradation by recognizing polyubiquitin chains. However, TYMS possess unique sequence motifs that enable ubiquitin-independent degradation [31]. Several studies reported that the N-terminal 45 amino acids of TYMS, comprising a 27-amino-acid intrinsically disordered region (IDR) followed by a highly conserved α-helix, function as an autonomous recognition motif (degron) for proteasomal degradation [32–36]. In cells, TYMS primarily functions as a homodimer, with its N-terminal residues forming an antiparallel interface at the dimerization site. This conformation preserves enzymatic activity while shielding TYMS from proteasomal degradation. Thus, TYMS stability is closely linked to its three-dimensional structure within the cell.

Post-translational modifications (PTMs) play a critical role in regulating protein stability and conformation. Two key PTMs implicated in TYMS dimer stabilization are acetylation and O-linked N-acetylglucosamine (O-GlcNAc) modification [34, 37]. To assess the impact of everolimus treatment on TYMS PTMs, we conducted immunoprecipitation assays. Notably, the acetylation of TYMS was not changed, and inhibition of deacetylases failed to restore TYMS protein levels (Fig. S5A, B).

However, O-GlcNAcylation of TYMS significantly decreased following everolimus exposure (T47D, *P* = 0.0093; MDA-MB-468, *P* = 0.0135) (Fig. 4D). Analysis of the GlyGen database identified O-GlcNAc modifications at T251 and T306, with N-acetylhexosamine (HexNAc) as the modified molecule. These modifications are known to lower the potential molecular energy of the TYMS monomer, enhance protein stability, and facilitate dimerization via hydrogen and π-σ bonding at the dimer interface [37]. To further examine these modifications, we performed non-labeled peptide quantitative analysis on everolimus-treated MDA-MB-468 cells using LC-MS, with an O-GlcNAcase inhibitor (Thiamet G, Th-G) present in all samples. This analysis identified two peptide sequences spanning T306, [293–308] and [288–308], both exhibiting T306-HexNAc modification (Fig. 4E, F). Importantly, the normalized abundance of the T306-HexNAc-modified 293-308 peptide was significantly lower in the everolimus-treated group (*P* = 0.0013), while the unmodified peptide abundance increased (*P* = 0.0111) (Fig. 4E). Although T306-HexNAc modification on the 288-308 peptide showed no significant difference, the non-modified peptide levels were elevated in the everolimus-treated group (*P* = 0.0338) (Fig. 4F). These findings indicate that everolimus markedly decreases O-GlcNAcylation of TYMS, leading to its destabilization and degradation.

To assess whether O-GlcNAcylation loss impacts TYMS dimer stability, we performed crosslinking experiments using disuccinimidyl suberate (DSS), a membrane-permeable, homo-bifunctional NHS ester. Notably, after 12 h of everolimus treatment, the TYMS monomer/dimer ratio increased (T47D, *P* = 0.019; MDA-MB-468, *P* = 0.0037), indicating enhanced TYMS dimer destabilization (Fig. S5C).

In summary, these findings demonstrate that everolimus reduces O-GlcNAcylation of TYMS, destabilizing its dimeric structure and exposing its degron, thereby downregulating TYMS protein levels and enhancing 5-FU sensitivity.

### mTORC1 inhibition reduces O-GlcNAc transferase expression and destabilizes thymidylate synthase in breast cancer

O-GlcNAcylation is a nutrient- and stress-responsive post-translational modification (PTM) [38] involving the attachment of O-linked N-acetylglucosamine (O-GlcNAc) moieties to serine (S) and threonine (T) residues in cytoplasmic, nuclear, and mitochondrial proteins. This modification is dynamically catalyzed by O-GlcNAc transferase (OGT) and removed by O-GlcNAcase (OGA) [39].

**Fig. 5:**
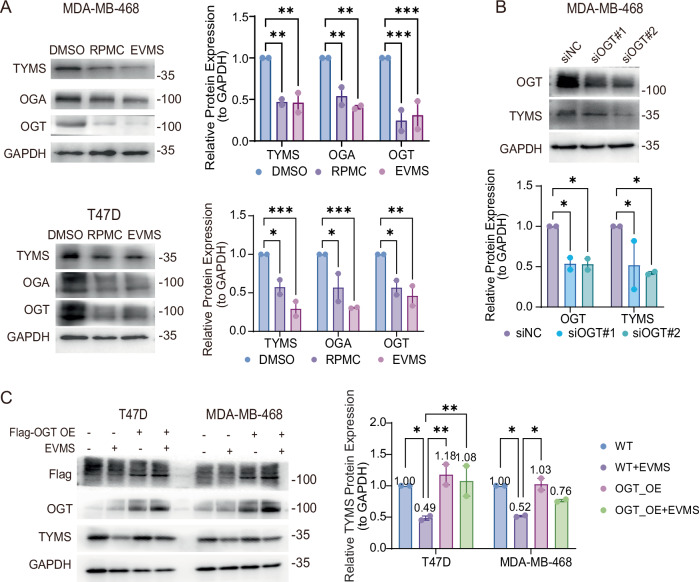
Everolimus destabilizes thymidylate synthase dimer in breast cancer cells via downregulation of O-GlcNAc transferase. **A** Western blot analysis and quantification of MDA-MB-468 and T47D cells treated with vehicle (DMSO), 10 nM rapamycin (RPMC), or 10 nM everolimus (EVMS) for 48 h. Densitometry values were normalized to GAPDH. Data are presented as mean ± SEM from two independent experiments and were analyzed by two-way ANOVA. **B** MDA-MB-468 cells were transfected with either negative control siRNA (siNC) or OGT siRNA (siOGT #1, siOGT #2) for 48 h. OGT and TYMS expression were evaluated by western blot. Quantification in both experiments is shown as mean ± SEM from two independent experiments, analyzed by two‑way ANOVA. **C** T47D and MDA-MB-468 cells were transfected with exogenous Flag‑tagged OGT and treated with or without 10 nM everolimus (EVMS) for 48 h. TYMS expression was then evaluated by western blot. Quantification in both experiments is shown as mean ± SEM from two independent experiments, analyzed by two‑way ANOVA. *P < 0.05; **P < 0.01; ***P < 0.001.

To determine how everolimus alters this balance and reduces TYMS O-GlcNAcylation, we analyzed mRNA and protein levels of OGT and OGA following everolimus and rapamycin treatment. Interestingly, OGT and OGA protein levels consistently declined after mTORC1 inhibition, independent of changes in transcriptomic expression (Figs. 5A and S5D). While treatment with an inhibitor of OGA, Thiamet-G, failed to restore TYMS levels after everolimus exposure (Fig. S5E), suggesting OGA is not involved in everolimus-mediated TYMS O-GlcNAcylation regulation.

To confirm OGT as a downstream effector of mTORC1 inhibition in TYMS regulation, we performed OGT knockdown in two breast cancer cell lines, which led to a reduction in TYMS levels (Figs. 5B and S5F). Furthermore, OGT overexpression did not alter basal TYMS levels, but restored TYMS levels reduced by everolimus (Fig. 5C), confirming its role in stabilizing TYMS.

These findings demonstrate that mTORC1 inhibition by everolimus disrupts OGT activity, leading to reduced O-GlcNAcylation of thymidylate synthase. This destabilization of its dimeric structure enhances proteasomal recognition, highlighting how everolimus, an oral targeted therapy, can potentiate the anti-cancer efficacy of the oral chemotherapy capecitabine in HER2-negative breast cancer. This mechanism offers valuable insights into developing future strategies to overcome resistance to breast cancer treatment (Fig. S5G).

### Everolimus and capecitabine exhibit synergistic anti-tumor activity in vivo

**Fig. 6:**
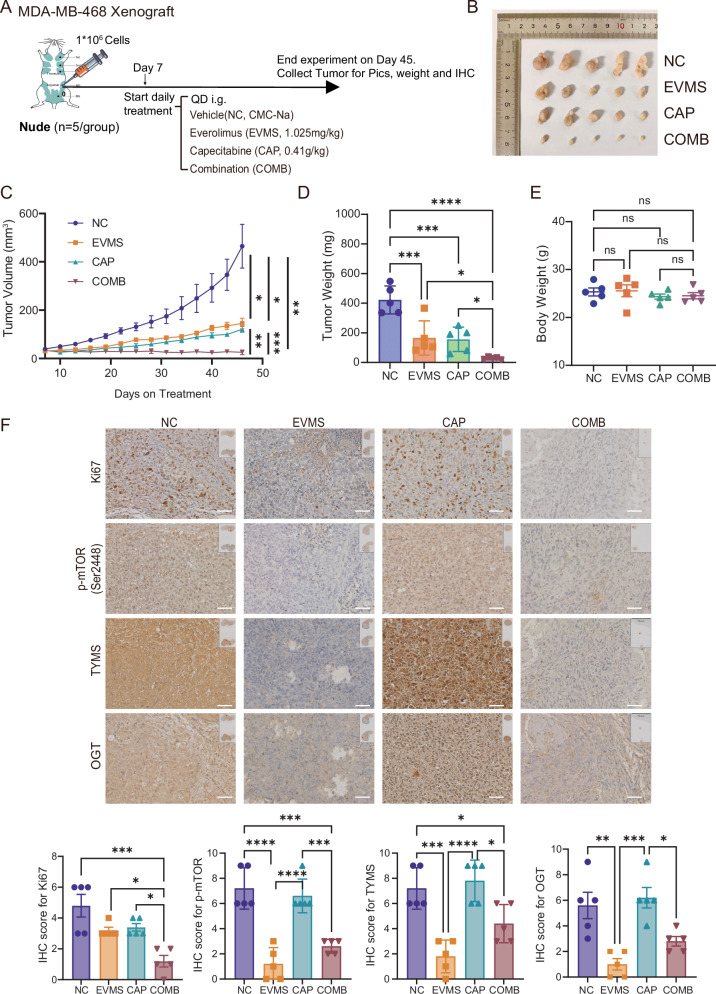
Everolimus and capecitabine exhibit synergistic anti-tumor activity in breast cancer in vivo. **A**–**D** In total, 1 × 10⁶ MDA-MB-468 cells were inoculated into the mammary fat pads of 6- to 8-week-old Balb/C Nude mice (*n* = 5 per group). When tumors became palpable (Day 7), mice were treated daily by oral gavage with 0.5% CMC-Na (NC), 1.025 mg/kg everolimus (EVMS), 0.41 g/kg capecitabine (CAP), or the combination EVMS + CAP (COMB). Tumor volumes (**C**) were measured every three days, and the experiment ended on Day 45. Mice were then euthanized, and tumors were collected for imaging and weighing (**B**, **D**). Data are shown as mean ± SEM, and statistical significance was determined by two-way ANOVA in (**C**) and one-way ANOVA in (**D**). **E** Endpoint body weight of MDA-MB-468 xenograft mouse models. Data are shown as mean ± SEM and analyzed by one-way ANOVA. **F** Representative images of Ki67, pS2448-mTOR, thymidylate synthase (TYMS), and O-GlcNAc transferase (OGT) immunohistochemical staining from tumors collected at the endpoint. Insets show navigator views. Scale bar is 50 μm. IHC scores are presented as bar graphs, and statistical analysis was performed by one-way ANOVA. Ns not significant; *P < 0.05; **P < 0.01; ***P < 0.001; ****P < 0.0001.

In vivo, MDA-MB-468 cells were orthotopically implanted into the fat pad of immunodeficient nude mice (aged 6–8 weeks), which were treated daily via oral gavage with vehicle (0.5% CMC-Na), everolimus (1.025 mg/kg, equivalent to a clinical dose of 5 mg/day), capecitabine (an oral 5-FU prodrug widely used in clinical practice, 0.41 g/kg, equivalent to 1 g BID), or a combination of both drugs. After 45 days, all mice were sacrificed and tumors collected (Fig. 6A, B). Both monotherapies exhibited tumor inhibition, but combination therapy exerted a significantly stronger suppressive effect (tumor volume, to EVMS, *P* = 0.003; to CAP, *P* = 0.0004; tumor weight, to EVMS, *P* = 0.0205; to CAP, *P* = 0.0291) (Fig. 6C, D). No significant differences in body weight were observed among groups (Fig. 6E), and no evident adverse effects occurred, confirming the safety and efficacy of combined treatment.

At the endpoint, the immunohistochemistry staining of Ki67 also showed a significant decrease in combination treatment group compared to vehicle and single agents (to NC, *P* = 0.0002; to EVMS, *P* = 0.0257; to CAP, *P* = 0.0135) (Figs. 6F and S6). Consistent with in vitro results, immunohistochemistry staining of MDA-MB-468 xenografts demonstrated that everolimus effectively inhibited mTOR phosphorylation, while capecitabine alone had no effect. Moreover, everolimus significantly reduced TYMS and OGT levels in tumor tissue, either alone or when combined with capecitabine (EVMS vs NC, *P* = 0.0002; COMB vs CAP, *P* = 0.0139) (Figs. 6F and S6). These results corroborate the in vitro mechanism and indicate that the combination of these two FDA-approved oral drugs produces synergistic anti-tumor efficacy in HER2-negative breast cancer.

## Discussion

In this study, we elucidate the synergistic effect between everolimus and fluorouracil (5-FU) in HER2-negative breast cancer. Mechanistically, mTORC1 inhibition enhances the cytotoxic efficacy of 5-FU by downregulating thymidylate synthase (TYMS) at the protein level, thereby reducing thymidine synthesis. This compensatory mechanism allows for lower chemotherapy doses while maintaining equivalent efficacy. We further identify TYMS as a promising sensitivity biomarker for this combination regimen. Although the role of mTORC1 in protein synthesis is well-documented, our findings reveal a novel function in regulating protein stability via post-translational modification (PTM). Specifically, mTORC1 inhibition suppresses O-GlcNAcylation by reducing O-GlcNAc transferase (OGT) levels, subsequently impairing TYMS dimerization and shortening its half-life. This study provides insight into non-canonical protein-degradation mechanisms, offering novel combination strategies using clinically approved drugs, which could significantly reduce translational costs.

The clinical impetus for this work is clear. The CREATE-X/JBCRG-04 Phase III trial demonstrated the efficacy of capecitabine in HER2-negative breast cancer patients, significantly improving survival outcomes following progression on first-line chemotherapy [1]. However, nearly 30% of these patients still relapse within 5 years, underscoring the critical need to prolong or expand fluoropyrimidine efficacy. While maximizing anti-cancer potency is a priority, patient adherence is equally vital. Oral medications like everolimus consistently achieve higher compliance, and its synergy with capecitabine represents a promising, patient-friendly option for advanced breast cancer that warrants further exploration [9, 10, 14, 40].

Our findings are situated within a well-established landscape of 5-FU resistance, a multifactorial phenomenon arising from mechanisms categorized into drug metabolism, transporter activity, DNA repair, tumor microenvironment, epigenetic changes, non-coding RNA imbalance, and dysregulated signaling pathways. A central and well-documented resistance axis is the overexpression or stabilization of TYMS [41, 42]. Consequently, strategies to destabilize TYMS, such as using curcumin [43] or the HDAC inhibitor trichostatin A [44] have been explored to resensitize tumors. This axis is complemented by other mechanisms, including transporter deficiencies (e.g., hENT1) [45] efflux pump upregulation (e.g., ABCB5) [46] non-coding RNA modulation [47, 48] and context-specific signaling pathway activation (e.g., PI3K/AKT) [49]. Our work directly extends this mechanistic landscape by identifying mTORC1 inhibition as a novel, clinically tractable means to destabilize TYMS via suppression of O-GlcNAcylation.

The pivotal role of TYMS in chemotherapy sensitivity is well-recognized [50]. SCAN-B RNA sequencing analysis revealed that mTORC1 signaling is highly enriched in TYMS-overexpressing breast cancer patients. Intriguingly, everolimus treatment downregulated TYMS independent of Ki67 expression. This destabilization is particularly relevant given that the reactive 5-FU metabolite FdUMP, while inactivating TYMS, paradoxically enhances its protein stability, a known contributor to resistance. Our finding that everolimus reverses TYMS overexpression induced by 5-FU treatment provides a direct rationale for its use in overcoming acquired resistance.

Our preliminary clinical evidence supports this rationale. We identified two heavily pretreated, refractory luminal B (HER2-negative) breast cancer patients who, after progressing on prior capecitabine, showed meaningful anti-tumor responses to the everolimus-capecitabine combination (Fig. S7A, B). One patient achieved the disappearance and shrinkage of metastatic lesions, while another exhibited a sustained decline in serum tumor markers, with no recorded treatment-related side effects. Furthermore, immunohistochemical analysis of paired tumor specimens from three additional patients pre- and post-everolimus-containing treatment revealed a significant downregulation of TYMS and OGT protein levels (Fig. S7C). These clinical observations provide a translational bridge, suggesting that everolimus induces TYMS and OGT downregulation in patients, supporting our proposed preclinical mechanism.

Emerging evidence highlights O-GlcNAcylation as a pivotal glycosylation modification in oncogenesis and therapy due to its high prevalence in cancer tissues [51, 52] regulation by only two conserved enzymes (OGT and OGA), and its role in nuclear/cytoplasmic processes like energy metabolism and stress response [52–55]. Targeting this axis via OGT downregulation is a promising therapeutic strategy linked to improved prognosis [56, 57]. However, effective direct OGT inhibitors remain elusive. Our research introduces the innovative possibility of using the clinically approved mTORC1 inhibitor everolimus to indirectly suppress O-GlcNAcylation, offering a novel and immediately actionable avenue to target this pathway.

The most critical next step emanating from this integrated body of work is to initiate prospective clinical trials. A primary objective must be the validation of TYMS as a predictive biomarker to stratify patients most likely to benefit. Successfully translating these findings holds the direct potential to benefit the substantial population of patients with refractory breast cancer.

In conclusion, this study provides a comprehensive understanding of how mTORC1 inhibition modulates TYMS stability via PTMs and O-GlcNAcylation, influencing chemotherapy sensitivity in breast cancer. The combination of everolimus and fluoropyrimidines presents a viable strategy to enhance treatment efficacy, overcome resistance, and improve patient adherence. Moreover, the indirect regulation of O-GlcNAcylation through mTORC1 inhibition offers an innovative approach to targeting cancer metabolism. These findings pave the way for new clinical trials exploring optimized therapeutic regimens, with the ultimate goal of reducing treatment costs and improving patient outcomes in advanced breast cancer management.

## Materials and methods

### Everolimus, 5-fluorouracil, and capecitabine preparation and treatment

Everolimus (RAD001, S1120), 5-fluorouracil (5-FU, S1209), and capecitabine (RO 09-1978, S1156) were purchased from Selleck. For in vitro studies, both everolimus and 5-fluorouracil were prepared as 10 mM stock solutions in DMSO and then diluted in complete culture medium immediately before cell treatment. Unless otherwise specified, cells were treated with 10 nM everolimus and 0.5 µM 5-FU in most in vitro experiments.

For in vivo experiments, everolimus was prepared as a 0.512 mg/mL stock solution (2× concentration for administration at 1.025 mg/kg), and capecitabine was prepared as a 0.205 g/mL stock solution (2× concentration for administration at 0.41 g/kg) in 0.5% CMC-Na (S6703; Selleck). The 2× drug solutions were freshly mixed together (or with 0.5% CMC-Na alone) immediately before treatment. Both drugs were administered once daily by oral gavage, and the in vivo drug stocks were freshly prepared every 3 days and stored at 4 °C.

### Cell culture, transfection, and establishment of stable cell lines

Breast cancer cell lines (MCF7, T47D, MDA-MB-231, BT549, MDA-MB-468, BT474, and SKBR3) and 293 T cells were obtained from the American Type Culture Collection (ATCC). All cell lines were authenticated by short tandem repeat (STR) profiling and confirmed to be mycoplasma-free. Cells were cultured in DMEM (Gibco, USA) supplemented with 10% fetal bovine serum (FBS; Hyclone, USA).

For siRNA transfection, cells were plated at 1 × 10⁵ cells per well in six-well plates and transfected with specific siRNAs (100 nM; GenePharma, China) using Lipofectamine 3000 (L3000150, Thermo Fisher) according to the manufacturer’s instructions. The siRNA sequences used were as follows:

- si-TYMS #1: GGGAUUCUCCACCAGAGAATT
- si-TYMS #2: GGAGUUGACCAACUGCAAATT
- si-OGT #1: GGAGGCAAUUGAGCAUUAUTT
- si-OGT #2: GAGCAGUAUUCCGAGAAAUTT

Successful transfections were validated by western blot.

HA-tagged TYMS (NM_001071) and FLAG-tagged OGT (NM_181672.3) were cloned into a pCDH-Puro vector (pCDH-CMV-MCS-EF1-GFP-T2A-Puro or pCDH-CMV-MCS-GFP-CMV-Puro). For lentivirus production, 6 µg of the pCDH construct was co-transfected into 293T cells along with 1 µg pCMV-VSVG and 3 µg pCMV-delta8.91 using Lipofectamine 3000. For transduction, T47D and MDA-MB-468 cells were plated at 1 × 10⁵ cells per well in 6-well plates and infected with lentiviral particles in the presence of 5 µg/mL Polybrene. After 2 days, puromycin (3 µg/mL) was added for selection of stable cell lines. Successful infections were confirmed by western blot.

### Dose–response, growth curve, and colony-formation assays

#### Dose–response curve

Cells were seeded at densities ranging from 1000 to 5000 cells per well in 96-well plates (depending on their doubling time) with six experimental replicates. Eight serially diluted concentrations (each concentration differing by a factor of two) were used for treatment. Treatment began 24 h after seeding and continued for 96 h. Cell viability was then assessed using the MTT assay. Briefly, MTT powder (3580MG250, Biofrox) was dissolved in sterile phosphate-buffered saline (PBS) at a concentration of 5 mg/mL and added to the cells at a 1:10 ratio. After a 4-h incubation at 37 °C, the supernatant was removed, the formazan crystals were dissolved in DMSO, and the absorbance was measured at 562 nm using a microplate spectrophotometer.

#### Growth curve

For growth analysis, cells were seeded into 96-well plates and treated as indicated. Cell viability was assessed by MTT assay at 24, 48, 72, and 96 h after treatment. The fold change in cell viability was determined by normalizing the absorbance on Day 4 to that on Day 1. These experiments were repeated at least three times.

#### Colony-formation assay

For colony-formation assays, T47D, MCF7, and MDA-MB-468 cells were seeded at 10,000, 5000, and 2000 cells per well, respectively, in six-well plates. Cells were cultured under the indicated treatments for 2 weeks. Colonies were fixed with 4% paraformaldehyde and stained with crystal violet. Colony counts and images were analyzed using vSpot Spectrum (AID). Colony-formation efficiency (CFE) was calculated as: CFE (%) = (colony number/seeding cell number) × 100%.

### Synergy assay

Cells were plated in 96-well plates arranged in a 7 × 6 or 6 × 6 matrix as shown in Figs. 1C and S1C–E. The drugs were added the next day, such that the dose of 5-FU in each well corresponded to the column header and the dose of everolimus corresponded to the row header. After 96 h of treatment, cell viability was measured using the MTT assay as described above. Wells in the first column (treated only with everolimus) and the first row (treated only with 5-FU) were used to establish the efficacy of every single agent, and various synergy scores (ZIP, Loewe, HSA, and Bliss) were calculated using the SynergyFinder+ web application [58]. The average synergy score across different drug concentrations is presented, with all calculations based on three biological replicates. Synergy score >20 indicating strong synergism, >10 synergy, 0–10 additive effects, and values < 0 antagonism.

### Flow cytometry for apoptosis and cell cycle

Flow cytometry was performed on a CytoFLEX S flow cytometer (Becton Dickinson) to analyze cell apoptosis and cell cycle distribution.

#### Apoptosis assay

For apoptosis analysis, the protocol of the Annexin V-FITC/PI Apoptosis Kit (40302ES60, YEASEN) was followed. Briefly, approximately 1 × 10^5^ cells were seeded in six-well plates, and treatment was initiated 24 h later. After 72 h of treatment, cells were trypsinized, stained with Annexin V-FITC for 15 min, and then with propidium iodide (PI) for 5 min. Apoptotic cells were defined as Annexin V-positive cells. Each experiment was performed with at least three biological replicates.

#### Cell cycle assay

About 1 × 10^6^ treated cells were fixed in 75% cold ethanol for at least 24 h. Before analysis, samples were washed with PBS to remove ethanol and then stained with 200 µL of 50 µg/mL PI and 10 µg/mL RNase in PBS for 30 min at room temperature. Data on the proportions of cells in the G0/G1, S, and G2/M phases were analyzed using ModFit LT 3.1.

#### EdU-based cell cycle assay

Cell lines were seeded into 10-cm dishes at densities of 0.5–2 × 10⁶ cells per dish, ensuring that the control group reached ~70% confluency by the end of the experiment. To label actively replicating DNA, EdU (5-ethynyl-2’-deoxyuridine, A10044, Invitrogen) was added to the culture media at a final concentration of 10 μM and incubated for 1 h at 37 °C before harvesting. Both floating and adherent cells were collected by harvesting media and trypsinization, followed by a single wash with cold PBS. Cells were fixed and permeabilized with ice-cold methanol and then stored at −20 °C overnight. The next day, methanol was removed by centrifugation at 1200 rpm for 2 min, followed by a PBS wash. The Click-iT reaction was performed following the BeyoClick™ EdU Cell Proliferation Kit with AF555 (C0075S, Beyotime) protocol, using Alexa Fluor™ 555 Azide for EdU detection. Cells were incubated with 500 μL of Click-iT reaction cocktail containing Alexa Fluor 555 azide at ambient temperature in the dark for 30 min. The reaction buffer was then removed by centrifugation at 1200 rpm for 2 min, followed by a PBS wash. Pellets were resuspended in 200 μL of 500 μg/ml RNase-containing DAPI solution (C0065, Solarbio) and incubated for 30 min at ambient temperature in the dark before flow cytometry analysis. Data were processed with CytExpert 2.5 software.

### EdU incorporation assay

The EdU proliferation assay was performed according to the instructions of the BeyoClick™ EdU Cell Proliferation Kit with AF555 (C0075S, Beyotime). Briefly, cells were seeded in 96-well plates at 5000 cells per well and treated with the indicated drugs for 48 h. In all, 10 µM EdU was added to the medium, and the cells were incubated for an additional 2 h at 37 °C. Cells were then fixed with 4% paraformaldehyde for 15 min and permeabilized with 0.1% Triton X-100 for 10 min, followed by one wash with PBS. Next, 50 µL of the Click-it reaction cocktail (prepared by mixing 43 µL Click Reaction Buffer, 2 µL CuSO₄, 0.1 µL Azide 555, and 5 µL Click-it additive solution) was added to each well, and the plate was incubated in the dark for 30 min at room temperature. After washing with PBS, cells were stained with Hoechst 33342 for 10 min before imaging. Images were captured using an IX71 fluorescence microscope (Olympus), and the number of EdU-positive cells was quantified using Fiji (ImageJ v1.53q).

### MDA-MB-468 xenograft mouse experiment

All animal experiments were approved by the South China University of Technology Laboratory Animal Center of SCUT (ACE: 2022053) in accordance with the guidelines. Six- to 8-week-old female Balb/C Nude mice were purchased from Hunan Slaek Jingda Experimental Animal Company. Mice were housed under specific pathogen-free conditions on a 12-h light/12-h dark cycle in temperature- and humidity-controlled cages, with food and water provided ad libitum.

Sample sizes were determined based on previous reports. Animals were randomly assigned to treatment or control groups. The investigators were blinded to group allocation during outcome assessments, including tumor volume, body weight, and histological evaluation. All collected data were included in the analyses, and no data were excluded.

For tumor implantation, 1 × 10^6^ MDA-MB-468 cells were suspended in 0.1 mL sterile PBS and orthotopically injected into the fourth mammary fat pad of each mouse. Once tumors became palpable (Day 7), the xenograft mice were randomly divided into four groups: 0.5% CMC-Na (NC); 1.025 mg/kg everolimus (EVMS); 0.41 g/kg capecitabine (CAP); EVMS + CAP (COMB).

Mice were treated once daily by oral gavage until Day 45. Tumor volume and body weight were measured every three days. On Day 45, all mice were euthanized, and tumors were collected for imaging, weighing, and further IHC staining.

### Immunohistochemistry (IHC) assay

Paraffin-embedded tissue sections were used for IHC staining. Slides were deparaffinized by heating at 65 °C for 1–2 h, followed by sequential immersion in xylene (2 × 10 min), graded ethanol (100%, 95%, 85%, 75%, 50%; each for 5 min), and distilled water (2 × 5 min). Antigen retrieval was then performed using either an acidic protocol, employing Sodium Citrate Antigen Retrieval Solution (50×; Beyotime, P0083), or an alkaline protocol using EDTA Antigen Retrieval Solution (pH 9.0; ZSGB-Bio, ZLI-9068) according to the primary antibody’s instructions. Slides were immersed in the retrieval buffer, subjected to high-pressure cooking for 3–5 min, and allowed to cool to room temperature.

Subsequently, the slides were incubated with 3% hydrogen peroxide solution (Boster, AR1108) for 10 min at room temperature to quench endogenous peroxidase activity, then blocked with 5% BSA (in PBS) for 30 min to minimize non-specific binding.

Primary antibodies, including TYMS (Proteintech, 15047-1-AP, 1:200), OGT (Proteintech, 11576-2-AP, 1:200), OGA (Proteintech, 14711-1-AP, 1:200), Ki67 (ZSGB-Bio, ZM-0167, 1:1), and pS2448-mTOR (Proteintech, 67778-1-1 G, 1:200), were diluted in 1% BSA and incubated overnight at 4 °C. After washing, the slides were incubated with the appropriate secondary antibodies (Peroxidase AffiniPure Goat Anti-Mouse IgG, Jackson ImmunoResearch, 115-035-003, 1:200; Peroxidase AffiniPure Goat Anti-Rabbit IgG, Jackson ImmunoResearch, 111-035-003, 1:200) for 1 h at room temperature. The signal was developed with DAB substrate solution (ZSGB-Bio, ZLI-9017) and counterstained with hematoxylin before dehydration in graded ethanol (70%, 85%, and 100%) and xylene. Representative images were captured using a Nikon ECLIPSE Ni-U microscope.

IHC staining scores were determined by two independent observers who evaluated 10 random fields per slide. The proportion of positive tumor cells was graded as follows: 0 (no positive cells), 1 (<10%), 2 (10–50%), and 3 (>50%). Staining intensity was scored as 0 (none), 1 (light brown), 2 (brown), and 3 (dark brown). The IHC score was calculated by multiplying the proportion score by the intensity score, resulting in scores of 0, 1, 2, 3, 4, 6, or 9.

### Real-time quantitative PCR with reverse transcription

Total RNA was extracted from breast cancer cells using the Total RNA Extraction Kit (ESscience, RN001). RNA was reverse transcribed into cDNA using the Hifair III 1st Strand cDNA Synthesis Super Mix for qPCR (gDNA Digester Plus Kit; YEASEN, 11141ES60). Real-time quantitative PCR was performed with Hieffi UNICON qPCR SYBR Green (YEASEN, 11198ES08) on a ProFlex PCR system (Thermo Fisher) according to the manufacturer’s recommendations using gene‑specific primers. Primer sequences were designed using Primer Bank (https://pga.mgh.harvard.edu/primerbank/). ACTB was used as the housekeeping gene, and relative gene expression was calculated using the 2^(–ΔΔCt) method.

The primer sequences used are as follows:

**Table:** 

Target	Forward (5’ → 3’)	Reverse(3’ → 5’)
DPYD	5’-ggcggacatcgagagtatcct-3'	5’-ttcttggccgaagtggaacac-3'
TYMS	5’-ggagtgaaaatctgggatgcc-3'	5’-actggaagccataaactgggc-3'
TYMP	5’-ggtgtgggtgacaaggtcag-3'	5’-gcagcacttgcatctgctc-3'
UMPS	5’-tctcgaccgcgtcttctga-3'	5’-acacacggtgtcaaaactgat-3'
TK1	5’-gccaaagacactcgctacag-3'	5’-cccctcgtcgatgcctatg-3'
OGA	5’-gaaggagagtcaagcgacgtt-3’	5’-tccataacccaaggtcttccat-3’
OGT	5’-tcctgatttgtactgtgttcgc-3'	5’-aagctactgcaaagttcggtt-3'
ACTB	5’-catgtacgttgctatccaggc-3'	5’-ctccttaatgtcacgcacgat-3'

### Western blotting

Proteins were extracted from cells using RIPA lysis buffer supplemented with protease and phosphatase inhibitors (78442, Thermo Scientific). Samples were separated by SDS-PAGE and transferred to polyvinylidene difluoride (PVDF) membranes. Membranes were incubated overnight at 4 °C with primary antibodies diluted 1:1000, followed by incubation with HRP-conjugated secondary antibodies (anti-mouse or anti-rabbit; 7076/7074, Cell Signaling Technology; diluted 1:5000) for 1 h at room temperature. The protein–antibody complexes were visualized using an enhanced chemiluminescence assay (34095, Pierce). All uncropped WB images were displayed in Extended File 1.

The antibodies used for western blotting are listed below:

**Table:** 

Antibodies	Brand	Catalog no.
Thymidylate Synthase (D5B3) XP® Rabbit mAb	Cell Signaling Technology	9045S
Thymidylate Synthase Mouse mAb	Proteintech	66725-1-IG
UMPS Rabbit pAb	Proteintech	14830-1-AP
TYMP Rabbit pAb	Abclonal	A1094
Thymidine Kinase 1 Rabbit mAb	Abclonal	A13612
DPYD Rabbit pAb	Proteintech	27662-1-AP
Anti-mouse O-GlcNAc Antibody	Santa Cruz	sc-59623
MGEA5 Rabbit pAb	Proteintech	14711-1-AP
OGT Rabbit mAb	Abclonal	A3501
HRP-conjugated GAPDH Mouse mAb	Proteintech	HRP-60004
Anti-mouse IgG HRP-linked Ab	Cell Signaling Technology	7076
Anti-rabbit IgG HRP-linked Ab	Cell Signaling Technology	7074
FLAG tag Mouse mAb	Proteintech	66008-4-lg

### Protein-degradation assay

T47D and MDA-MB-468 cells were treated with 100 µg/mL cycloheximide (CHX) to inhibit protein synthesis. Thymidylate synthase (TYMS) protein levels were measured by western blot at 0, 4, 8, and 12 h after CHX treatment. In parallel, cells were also treated with vehicle (DMSO) or 10 nM everolimus (EVMS) along with CHX. The reduction in TYMS levels over time represents the rate of protein degradation. Band intensities were normalized to GAPDH, and relative TYMS expression at each time point was calculated relative to the untreated baseline. The degradation kinetics were depicted using a line graph, and densitometric quantification was performed using ImageJ (v1.53q). All uncropped WB images are displayed in Extended File 1.

### Co-immunoprecipitation and ubiquitination assay

T47D and MDA-MB-468 cells were treated with 5 µM MG132 with or without 100 nM everolimus (EVMS) for 12 h. Cells from one 10 cm dish were harvested for each sample and lysed using Pierce IP Lysis Buffer (Thermo Fisher, 87787) supplemented with protease and phosphatase inhibitors. Ten percent of the lysate was set aside (mixed with 4× LDS Buffer, Thermo Fisher, NP0007) as the “input” control. The remaining lysate was incubated overnight at 4 °C with 5 µg of a specific antibody (including TYMS Rabbit pAb, Proteintech, 15047-1-AP; or Acetylated-Lysine Rabbit Antibody, CST, #9441) or with an irrelevant IgG (Rabbit IgG, Proteintech, B900610). Fresh Dynabeads were then added, and the mixture was incubated for an additional 1 h at 4 °C. Beads were washed with lysis buffer, and proteins were eluted in NuPAGE™ LDS Sample Buffer (4×) prior to analysis by SDS-PAGE. For the ubiquitination assay, an equivalent amount of whole-cell lysate was used as a positive control. Precipitated proteins were immunoblotted with total ubiquitin antibodies (Ubiquitin Mouse mAb, Immunoway, YM3636). Other primary antibodies for pulldown samples were as listed in the “Western blot” section.

### Intracellular protein–protein crosslinking experiment and TYMS monomer/dimer detection

T47D and MDA-MB-468 cells were treated with 5 µM MG132 with or without 100 nM everolimus (EVMS) for 12 h. After trypsinization, cells were washed three times with ice-cold PBS and resuspended at a final concentration of 25 × 10⁶ cells/mL. Disuccinimidyl suberate (DSS; Selleck, S6929), a membrane-permeable bifunctional crosslinker, was dissolved in DMSO to prepare a 25 mM stock solution and then added to the cell suspension to a final concentration of 5 mM. The reaction was carried out on a shaker at room temperature for 1 h, followed by the addition of a 10× glycine quencher (Thermo Fisher, 7005) and incubation at room temperature for 15 min. The cells were then centrifuged at 300× *g* for 3 min to remove the crosslinking solution, washed twice with PBS, and lysed using Pierce IP Lysis Buffer with inhibitors. Subsequent steps were conducted following standard western blot procedures. Thymidylate Synthase (D5B3) XP® Rabbit mAb (Cell Signaling Technology, 9045S) was used to detect both the dimer (70 kDa) and monomer (35 kDa) forms of TYMS. The relative monomer/dimer ratio was calculated via densitometric analysis using ImageJ.

### Mass spectrometry

Mass spectrometry analysis was performed by the Bioinformatics and Omics Center at Sun Yat-Sen Memorial Hospital, Sun Yat-Sen University (Guangzhou, China). Briefly, MDA-MB-468 cells treated with 2 µM Thiamet-G and vehicle (DMSO) or 100 nM everolimus for 12 h were harvested and sonicated to extract peptides. A total of 300 µg protein was diluted in 50 mM NH₄CO₃ to 100 µL and precipitated overnight at –20 °C with cold acetone. Reduction was performed using 1 M dithiothreitol (DTT) followed by alkylation with 500 mM iodoacetamide (IAA). Trypsin digestion was carried out at an enzyme-to-protein ratio of 1:40–1:50 at 37 °C for 12–18 h. The reaction was stopped with 0.4% trifluoroacetic acid (TFA), and peptides were desalted using solid phase extraction (SPE) columns, washed with TFA buffer, and eluted with an acetonitrile-based buffer. The eluate was vacuum-dried, stored at –80 °C, and reconstituted in 0.1% formic acid. After vortexing and centrifugation, the supernatant was collected, and the peptide concentration was determined before LC-MS analysis using an Orbitrap Fusion Mass Spectrometer with a Nano electrospray ion source (Thermo Fisher Scientific). Data were analyzed using Proteome Discoverer 2.4 for label-free quantification (LFQ-MS). Collision-induced dissociation (CID) spectra were acquired with SequestHT and validated with Percolator. Peptide sequences were searched against the human proteome FASTA database with a precursor mass tolerance of 10ppm, a fragment mass tolerance of 0.6 Da, and up to 2 missed cleavages allowed. Variable dynamic modifications included oxidation (M), phosphorylation (S, Y), HexNAc (N, S, T), methylation (D, E, H, I), and glycosylation (T), while carbamidomethylation (C) was set as a fixed modification. Targeted measurements of intracellular metabolites were also performed, and relative metabolite abundance was quantified using integrated peak areas from multiple-reaction-monitoring transitions. Data represent three replicates per condition.

### Breast cancer patients database analysis

#### TYMS expression analysis in normal and cancerous breast tissue

mRNA expression levels of TYMS were obtained from the GTEx and TCGA datasets [59] to compare normal breast tissue with breast cancer tissue. Data processing and normalization were performed via the online website: targeted prognostic analysis module | bc-GenExMiner using standard bioinformatics pipelines, and statistical analyses were conducted to assess differences in TYMS expression.

#### Prognostic analysis of TYMS in breast cancer

TYMS expression data and overall survival information were extracted from the SCAN-B and METABRIC databases as well as from the Affymetrix platform [60]. Kaplan–Meier survival curves were generated using the online bioinformatic website: targeted prognostic analysis module | bc-GenExMiner to evaluate the prognostic significance of TYMS in breast cancer. Log-rank tests were used to assess significance, and the median TYMS expression level was employed as the cutoff. Kaplan–Meier plots for different breast cancer subtypes were generated using TCGA data [61] with the optimal cutoff automatically selected by the online Kaplan–Meier Plotter: Kaplan-Meier plotter [Breast RNAseq GSE96058].

#### Gene set enrichment analysis (GSEA)

Breast cancer patients from the SCAN-B database [62] were stratified into the top 10 and bottom 10 TYMS-expressing groups based on mRNA FPKM levels (GSE96058). Transcriptomic data from these groups were analyzed using GSEA 4.3.0 software with gene sets curated from MSigDB_Hallmarks to identify significantly enriched pathways using a false discovery rate (FDR) threshold.

#### Single-sample gene set enrichment analysis (ssGSEA)

ssGSEA was performed using log₂-transformed TPM data from 5-FU treated TCGA cohort. Enrichment scores for mTORC1_signalling gene sets were calculated with the GSVA package. And differentially expressed genes (adj *P* < 0.05, |Log2FC | > 1) were indicated on the heatmap.

### Study cohort and specimen collection

This study utilized archived formalin-fixed, paraffin-embedded (FFPE) tumor specimens from 3 female breast cancer patients treated at the Breast Tumor Center of Sun Yat-Sen Memorial Hospital. All patients in this cohort received everolimus as part of their therapeutic regimen. Tumor biopsies were obtained either before or after the initiation of everolimus treatment, and three patients provided paired pre- and post-treatment samples.

### Ethics statement and data collection

The study protocol and design were reviewed and approved by the Institutional Review Board and Ethics Committee of Sun Yat-Sen Memorial Hospital (approval number: SYSKY-2025-886-01) in accordance with the guidelines for Good Clinical Practice and the Declaration of Helsinki. The collection of all human specimens and associated clinical data was conducted with written informed consent from each patient. Patient treatment history and hospital examination data were retrieved from medical records; these data reflect standard procedures for tumor burden monitoring during routine clinical care, and no additional tests were conducted for the purpose of this study.

### Statistical analysis

Data were analyzed using GraphPad Prism (version 10.3.1). Parametric tests were applied as all data met assumptions of normality and equal variance. All in vitro and animal experiment results are expressed as mean ± SEM. For comparisons between two groups, an unpaired two-tailed Student’s *t* test was used. For comparisons among more than two groups, one-way ANOVA followed by Dunnett’s multiple comparisons test was used when comparing each treatment group with a single control. For all pairwise comparisons, two-way ANOVA followed by Tukey or Šidak multiple comparisons test was performed. For dose–response curve, cell viability was fitted with drug dose in a nonlinear regression model (dose-normalized-response inhibition). IC50 values were calculated by GraphPad and compared as unshared parameters by extra sum-of-squares *F* test (*P < 0.05, **P < 0.01, ***P < 0.001, ****P < 0.0001). Survival curves were compared using the log-rank (Mantel–Cox) test. Western blot band intensity was quantified using ImageJ. Sample size and specific statistical methods used for each analysis are provided in the figure legends where applicable.

## Supplementary information

Supplementary Figures S1-S7

Extended File 1 Uncropped WB image

## Data Availability

All data generated or analyzed during this study are included in this published article and its supplementary information files.
