# Supplementary material for: Everolimus destabilizes thymidylate synthase via suppressing its O-GlcNAcylation and sensitizes HER2-negative breast cancer to fluorouracil
Source: Cell Death Dis. 2026 Apr 4;17(1):456. doi: 10.1038/s41419-026-08715-z (PMC13184257; doi:10.1038/s41419-026-08715-z)
Supplement: Supplementary file 1 — Supplementary Figures S1-S7 [file 41419_2026_8715_MOESM1_ESM.pdf]

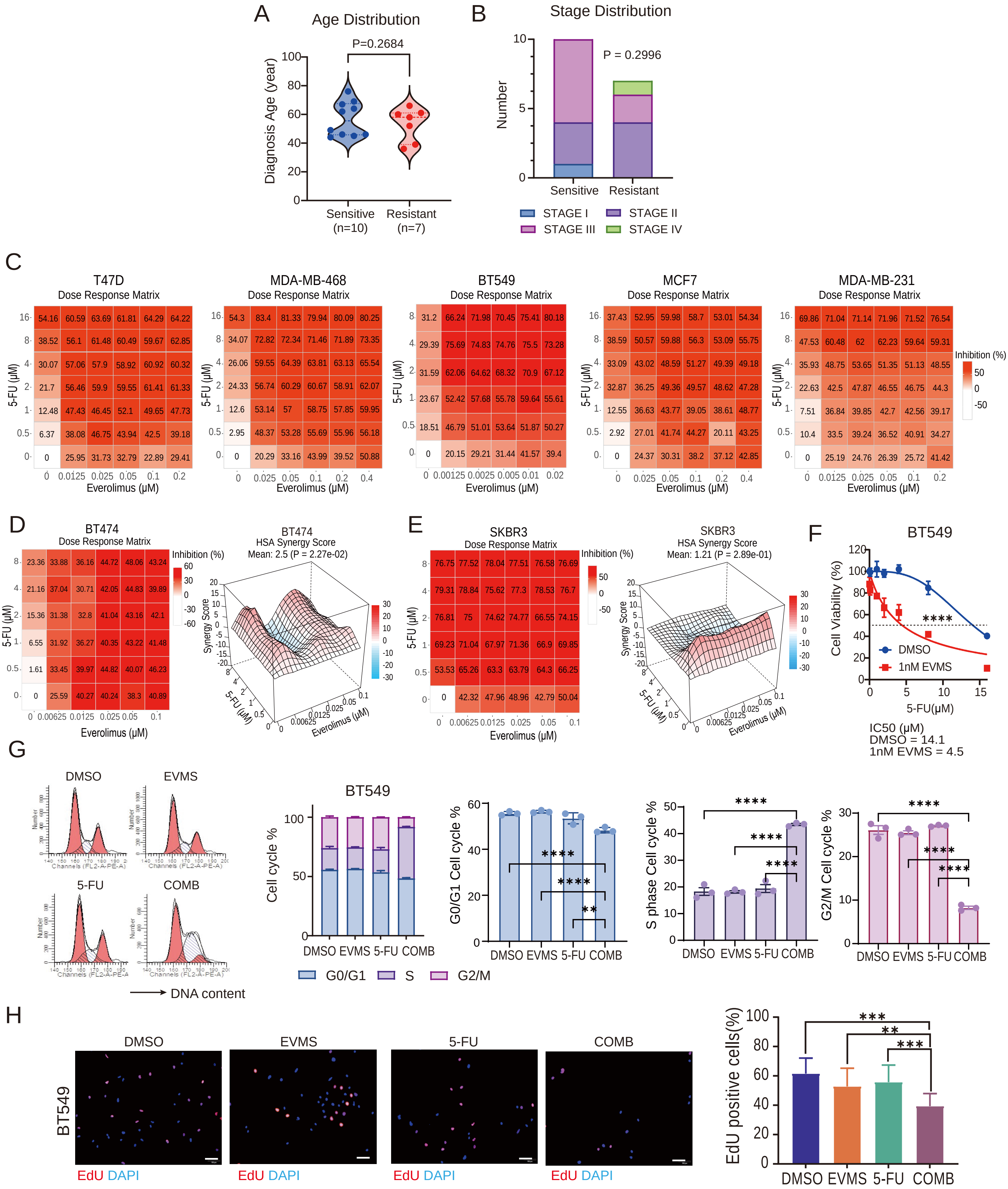
A
Age Distribution
P=0.2684
Diagnosis Age (year)
Sensitive (n=10)
Resistant (n=7)
B
Stage Distribution
P = 0.2996
Number
Sensitive
Resistant
STAGE I
STAGE II
STAGE III
STAGE IV
C
T47D Dose Response Matrix
MDA-MB-468 Dose Response Matrix
BT549 Dose Response Matrix
MCF7 Dose Response Matrix
MDA-MB-231 Dose Response Matrix
5-FU (μM)
Everolimus (μM)
Inhibition (%)
D
BT474 Dose Response Matrix
BT474 HSA Synergy Score
Mean: 2.5 (P = 2.27e-02)
Synergy Score
E
SKBR3 Dose Response Matrix
SKBR3 HSA Synergy Score
Mean: 1.21 (P = 2.89e-01)
F
BT549
Cell Viability (%)
****
DMSO
1nM EVMS
5-FU(μM)
IC50 (μM)
DMSO = 14.1
1nM EVMS = 4.5
G
DMSO
EVMS
5-FU
COMB
Channels (FL2-A-PE-A)
DNA content
BT549
Cell cycle %
G0/G1 Cell cycle %
S phase Cell cycle %
G2/M Cell cycle %
G0/G1
S
G2/M
H
DMSO
EVMS
5-FU
COMB
BT549
EdU DAPI
EdU positive cells(%)

fig. S1 Everolimus increases HER2-negative breast cancer susceptibility to fluoropyrimidine. (Related to Fig. 1)

(A) Diagnosis age distribution of patients from Fig. 1A. Statistical significance was assessed by unpaired Student's t test.

(B) Breast cancer AJCC TNM Stage distribution from Fig. 1A. Statistical significance was assessed by Fisher's exact test.

(C) Dose response matrix of Fig. 1C.

(D-E) Dose response matrix and HSA synergy score 3D heatmaps of everolimus (EVMS) and 5-fluorouracil (5-FU) combination treatment in HER2-positive **(D)** BT474, **(E)** SKBR3 breast cancer cell lines

(F) Dose-response curve of 5-fluorouracil (5-FU) in BT549 cells treated with either vehicle (DMSO) or 1 nM everolimus (EVMS) for 96 hours. Dose-response curves were fitted using a nonlinear regression model. Data were presented as mean ± SEM from three independent experiments and compared as unshared parameters by Extra sum-of-squares F test.

(G) Cell cycle analysis of BT549 cells treated with vehicle (DMSO), 10 nM everolimus, 0.5 μM 5-FU, or their combination for 48 hours. Data are shown as mean ± SEM from three independent experiments, and significance was determined by two-way ANOVA.

(H) EdU incorporation assay in BT549 cells treated with vehicle (DMSO), 10 nM everolimus, 0.5 μM 5-FU, or their combination for 48 hours. Data are presented as mean ± SEM from three independent experiments and were analyzed by one-way ANOVA. The scale bar represents 100 μm. $^{**}P < 0.01$; $^{***}P < 0.001$; $^{***}P < 0.0001$.

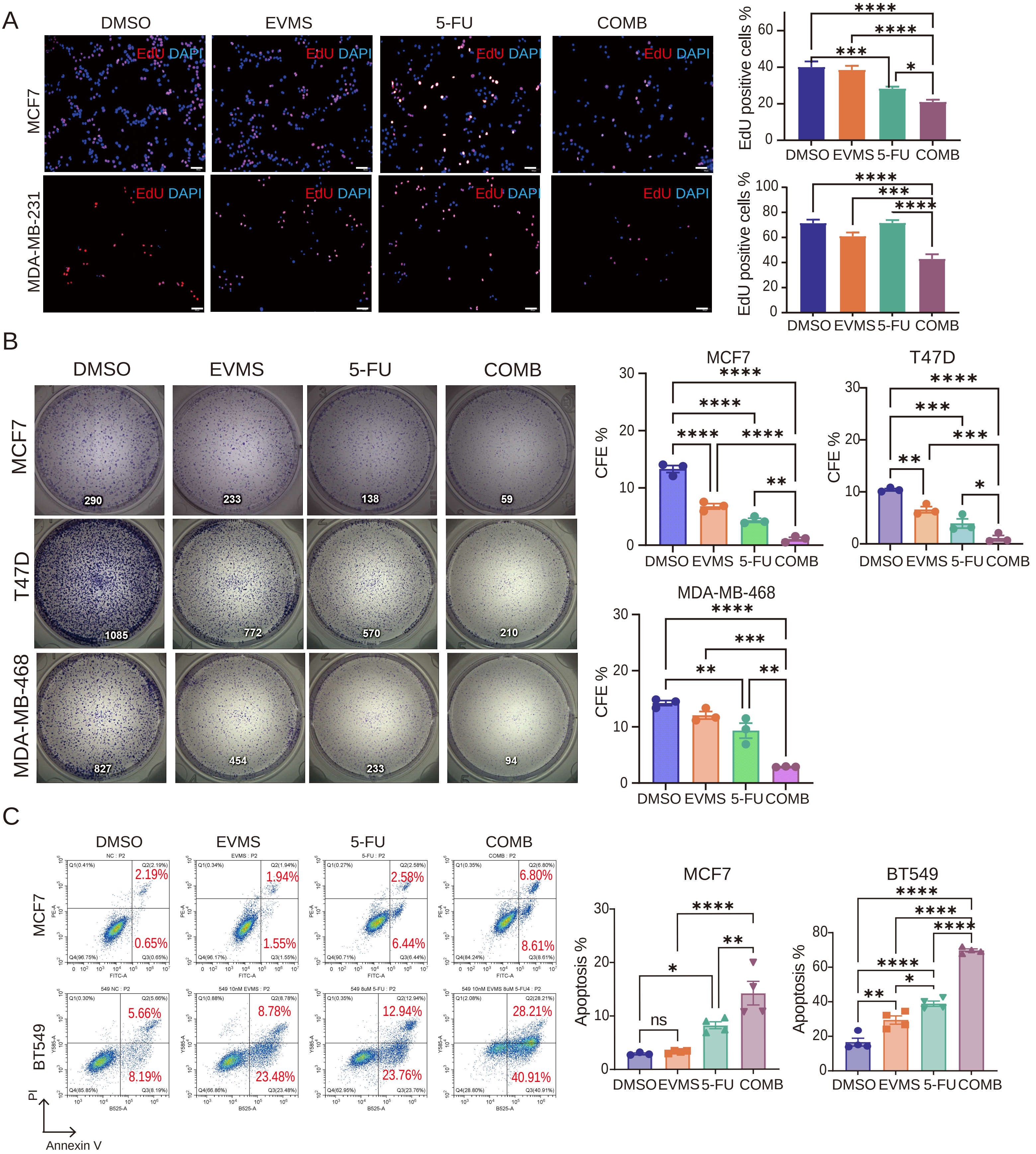

A
DMSO
EVMS
5-FU
COMB
MCF7
MDA-MB-231
EdU DAPI
EdU positive cells %
DMSO EVMS 5-FU COMB
B
MCF7
T47D
MDA-MB-468
290
233
138
59
1085
772
570
210
827
454
233
94
CFE %
C
2.19%
0.65%
1.94%
1.55%
2.58%
6.44%
6.80%
8.61%
5.66%
8.19%
8.78%
23.48%
12.94%
23.76%
28.21%
40.91%
BT549
PI
Annexin V
Apoptosis %
ns

fig. S2 Everolimus increases HER2-negative breast cancer susceptibility to fluoropyrimidine. (Related to Fig. 1)

(A) EdU incorporation assay in MCF7 and MDA-MB-231 cells treated with vehicle (DMSO), 10 nM everolimus, 0.5 μM 5-FU, or their combination for 48 hours. Data are presented as mean ± SEM from three independent experiments and were analyzed by one-way ANOVA. The scale bar represents 100 μm.

(B) Representative images and quantification of colony formation assays in MCF7, T47D and MDA-MB-468 cells treated with vehicle (DMSO), 10 nM everolimus (EVMS), 0.5 μM 5 fluorouracil (5 FU), or their combination (COMB). The colony numbers indicated on each image correspond to the following initial seeding numbers: MCF7, 5000; T47D, 10000; MDA-MB-468, 2000 cells per well. Data are shown as mean ± SEM from three independent experiments, and statistical significance was determined by one-way ANOVA. The scale bar represents 100 μm.

(C) Apoptosis assay in MCF7 and BT549 cells treated with vehicle (DMSO), 10 nM everolimus, 0.5 μM 5 FU, or their combination for 72 hours. The bar graph depicts the percentage of Annexin V positive cells. Data are expressed as mean ± SEM from four independent experiments and were analyzed using one-way ANOVA. Ns, not significant; **P* < 0.05; ***P* < 0.01; ****P* < 0.001; *****P* < 0.0001.

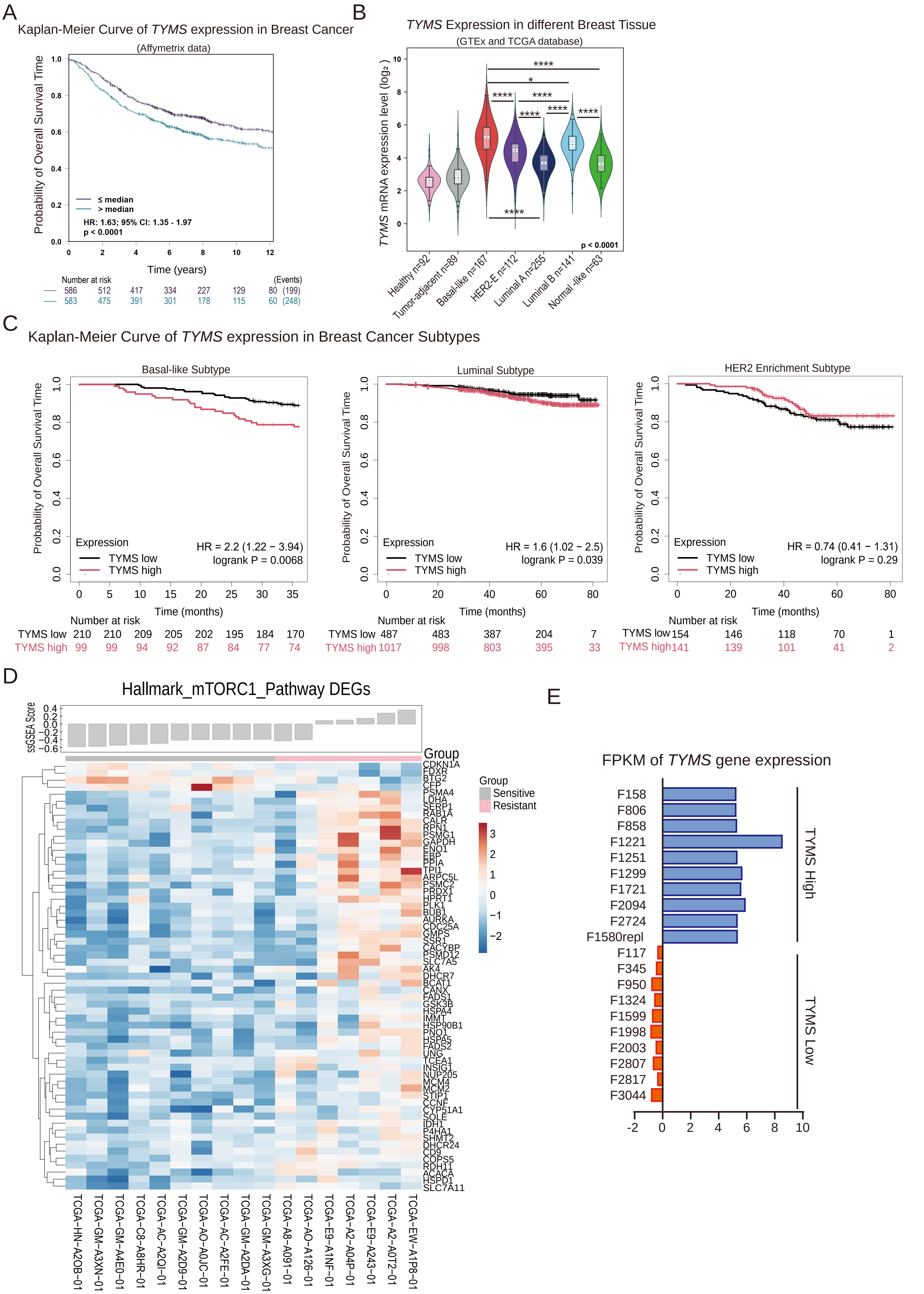

A
Kaplan-Meier Curve of TYMS expression in Breast Cancer
(Affymetrix data)
Probability of Overall Survival Time
Time (years)
≤ median
> median
HR: 1.63; 95% CI: 1.35 - 1.97
p < 0.0001
Number at risk (Events)
586 512 417 334 227 129 80 (199)
583 475 391 301 178 115 60 (248)
B
TYMS Expression in different Breast Tissue
(GTEx and TCGA database)
TYMS mRNA expression level (log₂)
p < 0.0001
Healthy n=92
Tumor-adjacent n=89
Basal-like n=167
HER2-E n=112
Luminal A n=255
Luminal B n=141
Normal -like n=63
C
Kaplan-Meier Curve of TYMS expression in Breast Cancer Subtypes
Basal-like Subtype
Luminal Subtype
HER2 Enrichment Subtype
Probability of Overall Survival Time
Time (months)
Expression
TYMS low
TYMS high
HR = 2.2 (1.22 − 3.94)
logrank P = 0.0068
HR = 1.6 (1.02 − 2.5)
logrank P = 0.039
HR = 0.74 (0.41 − 1.31)
logrank P = 0.29
Number at risk
TYMS low 210 210 209 205 202 195 184 170
TYMS high 99 99 94 92 87 84 77 74
TYMS low 487 483 387 204 7
TYMS high 1017 998 803 395 33
TYMS low 154 146 118 70 1
TYMS high 141 139 101 41 2
D
Hallmark_mTORC1_Pathway DEGs
ssGSEA Score
Group
Sensitive
Resistant
CDKN1A FDXR BTG2 CFP PSMA4 LDHA SERP1 RAB1A CALR RPN1 PSMG1 GAPDH ENO1 EBP PPIA TPI1 ARPC5L PSMC2 PRDX1 HPRT1 PLK1 BUB1 AURKA CDC25A GMPS SSR1 CACYBP PSMD12 SLC7A5 AK4 DHCR7 BCAT1 CANX FADS1 GSK3B HSPA4 IMMT HSP90B1 PNO1 HSPA5 FADS2 UNG TCEA1 INSIG1 NUP205 MCM4 MCM2 STIP1 CCNF CYP51A1 SQLE IDH1 P4HA1 SHMT2 DHCR24 CD9 COPS5 RDH11 ACACA HSPD1 SLC7A11
TCGA−HN−A2OB−01 TCGA−GM−A3XN−01 TCGA−GM−A4E0−01 TCGA−C8−A8HR−01 TCGA−AC−A2QI−01 TCGA−GM−A2D9−01 TCGA−AO−A0JC−01 TCGA−AC−A2FE−01 TCGA−GM−A2DA−01 TCGA−GM−A3XG−01 TCGA−A8−A091−01 TCGA−AO−A126−01 TCGA−E9−A1NF−01 TCGA−A2−A04P−01 TCGA−E9−A243−01 TCGA−A2−A0T2−01 TCGA−EW−A1P8−01
E
FPKM of TYMS gene expression
F158 F806 F858 F1221 F1251 F1299 F1721 F2094 F2724 F1580repl
F117 F345 F950 F1324 F1599 F1998 F2003 F2807 F2817 F3044
TYMS High
TYMS Low

fig. S3 Thymidylate synthase is a prognostic biomarker and predictive target for combination therapy in HER2-negative breast cancer. (Related to Fig. 2)

(A) Kaplan–Meier survival curves for overall survival analysis of breast cancer patients stratified by *TYMS* expression, derived from an Affymetrix database. The cutoff for *TYMS* was set at the median value. Hazard ratios (HR) with 95% confidence intervals and log-rank P values are provided.

(B) Violin plots showing TYMS expression in healthy breast tissue, tumor adjacent tissue, and various breast cancer subtypes. Data was obtained from the GTEx and TCGA databases.

(C) Kaplan–Meier survival curves for overall survival analysis of Basal-like, luminal, or HER2-enriched breast cancer patients with varying levels of TYMS expression. In this analysis, the optimal cutoff for TYMS expression was selected. Hazard ratios (HR) with 95% confidence intervals and log-rank P values are shown.

(D) Bar plot of ssGSEA scores of Hallmark_mTORC1_signalling pathways across patients from Fig. 1A and heatmap of DEGs (adj $p < 0.05$, $|log2FC| > 1$) from this pathway.

(E) The top 10 and bottom 10 breast cancer patients based on *TYMS* expression from the SCAN-B database (GSE96058) were grouped as TYMS-high and TYMS-low, respectively. The X-axis shows the FPKM levels of TYMS in these 20 samples, and the Y-axis lists each sample name. **P* < 0.05; *****P* < 0.0001.

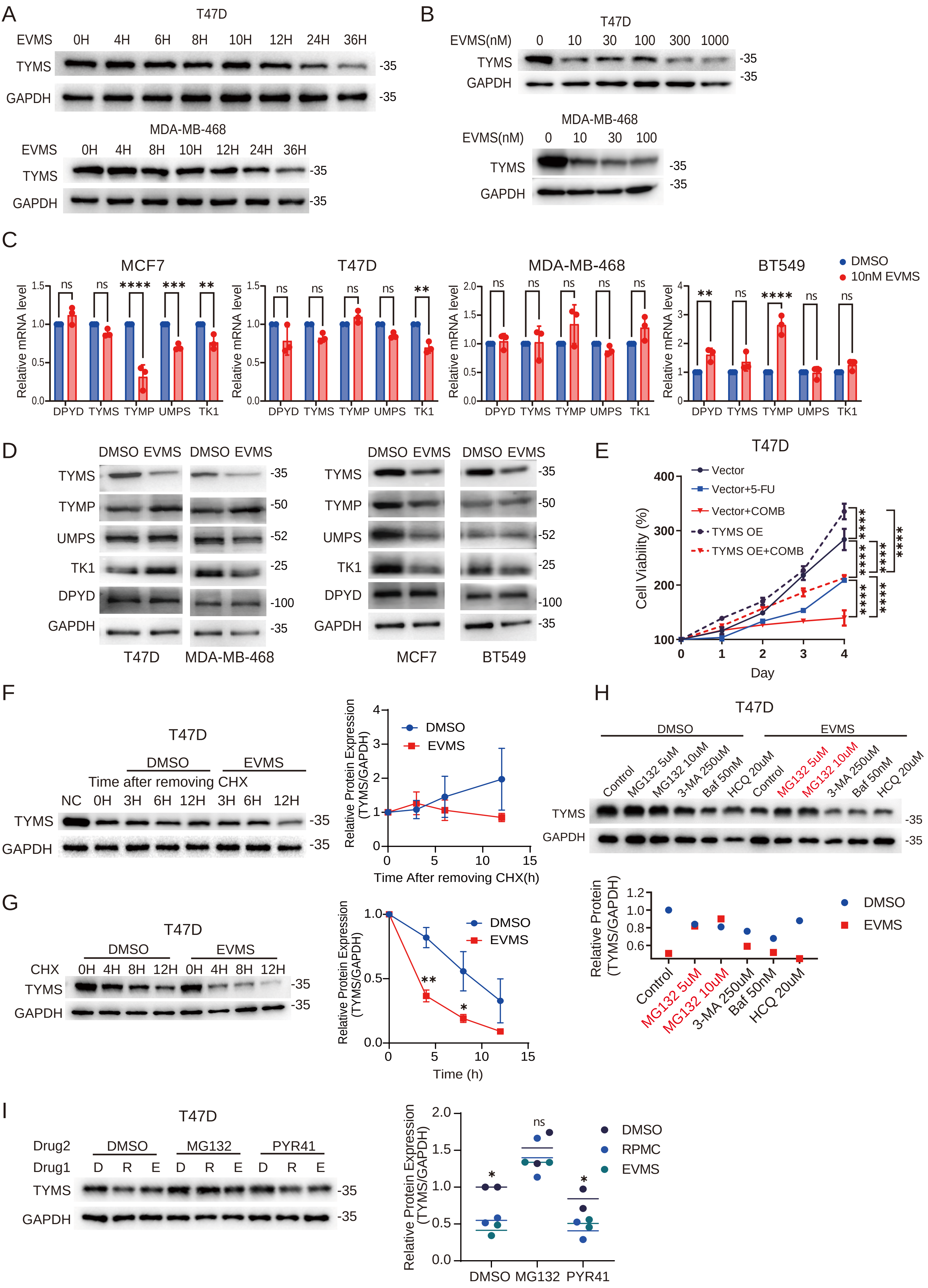
A
T47D
EVMS 0H 4H 6H 8H 10H 12H 24H 36H
TYMS
GAPDH
-35
MDA-MB-468
EVMS 0H 4H 8H 10H 12H 24H 36H
B
T47D
EVMS(nM) 0 10 30 100 300 1000
MDA-MB-468
EVMS(nM) 0 10 30 100
C
MCF7
T47D
MDA-MB-468
BT549
Relative mRNA level
DPYD TYMS TYMP UMPS TK1
DMSO
10nM EVMS
ns
D
DMSO EVMS DMSO EVMS
TYMS
TYMP
UMPS
TK1
DPYD
GAPDH
-35 -50 -52 -25 -100
T47D MDA-MB-468 MCF7 BT549
E
T47D
Vector
Vector+5-FU
Vector+COMB
TYMS OE
TYMS OE+COMB
Cell Viability (%)
Day
F
T47D
DMSO EVMS
Time after removing CHX
NC 0H 3H 6H 12H 3H 6H 12H
Relative Protein Expression (TYMS/GAPDH)
Time After removing CHX(h)
G
T47D
CHX 0H 4H 8H 12H 0H 4H 8H 12H
Time (h)
H
T47D
Control MG132 5uM MG132 10uM 3-MA 250uM Baf 50nM HCQ 20uM
Relative Protein (TYMS/GAPDH)
I
T47D
Drug2 DMSO MG132 PYR41
Drug1 D R E D R E D R E
DMSO
RPMC
EVMS
DMSO MG132 PYR41

fig. S4 Everolimus enhances fluorouracil sensitivity in breast cancer via thymidylate synthase abrogation. (Related to Fig. 3-4)

(A-B) Western blot analysis of the indicated cell lines under various conditions: (**A)** Treatment with 10 nM everolimus (EVMS) for the indicated time points; **(B)** Treatment with different doses of everolimus for 24 hours.

(C) qPCR analysis of mRNA expression levels of thymidylate synthase (TYMS), thymidine phosphorylase (TYMP), uridine monophosphate synthetase (UMPS), thymidine kinase 1 (TK1) and dihydropyrimidine dehydrogenase (DPYD) in MCF7, T47D, MDA-MB-468, and BT549 cells treated with vehicle (DMSO) or 10 nM everolimus (EVMS) for 48 hours. ACTB was used as the reference gene, and relative gene expression was calculated using the $2^{(-\Delta\Delta Ct)}$ method. Data were presented as mean ± SEM from three independent experiments and analyzed by one-way ANOVA.

(D) Western blot analysis of DPYD, TYMS, TYMP, UMPS, and TK1 in MCF7, T47D, MDA-MB-468, and BT549 cells treated with vehicle (DMSO) or 10 nM everolimus (EVMS) for 48 hours.

(E) Growth curve analysis of control (Vector) or TYMS-overexpressing T47D cells treated with vehicle, 0.5 µM 5-fluorouracil (5-FU), or the combination. Data from three independent experiments are shown as mean ± SEM, and significance was determined by two-way ANOVA on Day 4.

(F) TYMS synthesis assay. Western blot analysis and quantification of T47D cells treated with cycloheximide (CHX, 25 µg/ml) for 18 hours, followed by replacement with either vehicle (DMSO) or 10 nM everolimus (EVMS) for the indicated times. Relative protein expression was normalized to the 0-hour time point after CHX removal. Data from two independent experiments are shown as mean ± SEM.

(G) T47D were treated with 100 µg/ml cycloheximide (CHX) and either vehicle (DMSO) or 10 nM everolimus (EVMS) for the indicated time points. Data from two independent experiments are shown as mean ± SEM, and significance was determined using two-way ANOVA.

(H) Western blot analysis and quantification of T47D cells under the indicated treatments for 12 hours. Treatment with 10 nM everolimus (EVMS) was used to downregulate TYMS levels to elucidate the dominant degradation pathway.

(I) Western blot analysis and quantification of T47D cells treated with DMSO (D), 100 nM rapamycin (R), or 100 nM everolimus (E) along with DMSO, 5 µM MG132, and 5 µM PYR-41 for 12 hours. Densitometry was normalized to GAPDH and no treatment group. Data from two independent experiments are presented as mean ± SEM, and analysis was performed using two-way ANOVA. Ns, not significant; $^{*}P < 0.05$; $^{**}P < 0.01$; $^{***}P < 0.001$; $^{****}P < 0.0001$.

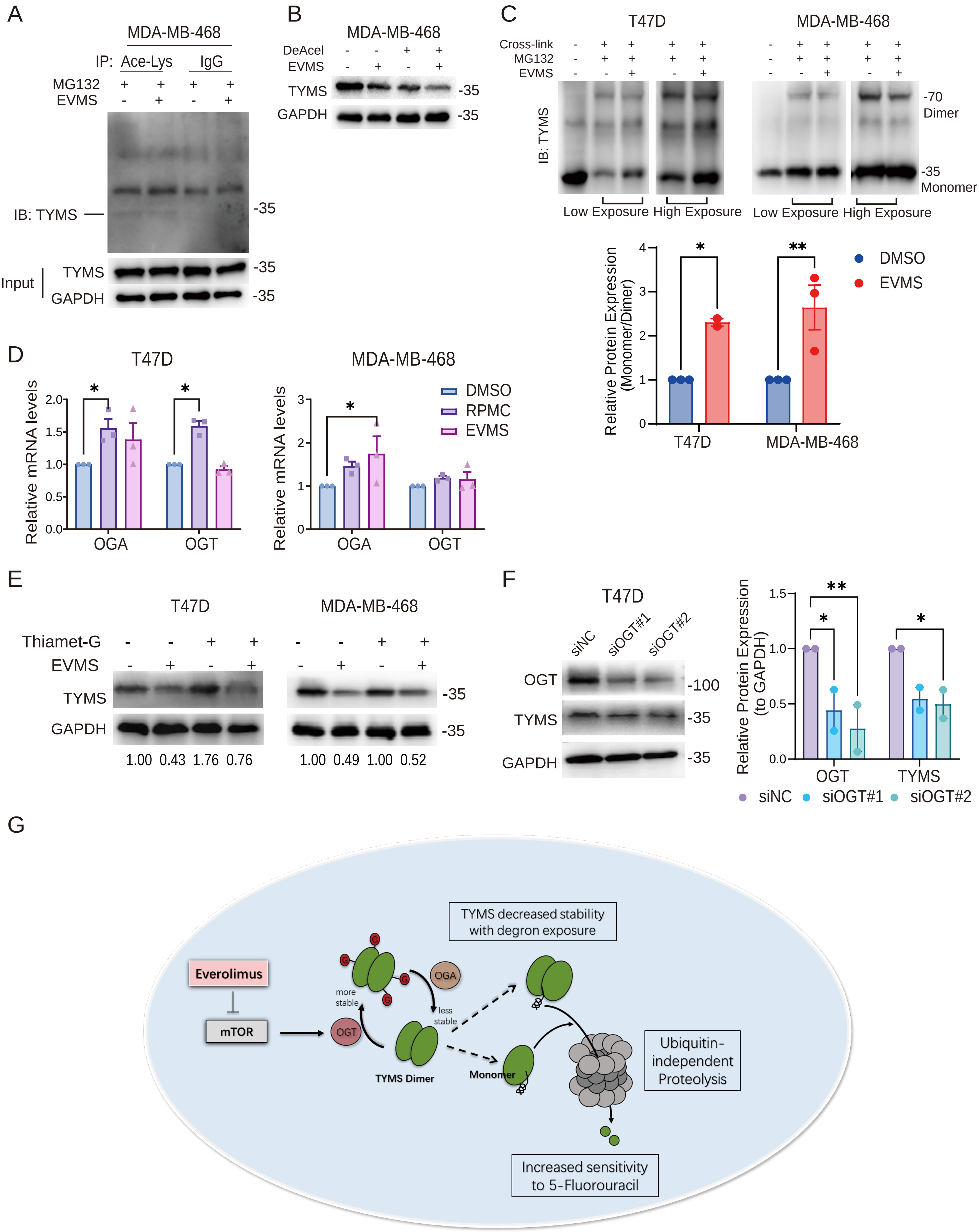

A
MDA-MB-468
IP: Ace-Lys IgG
MG132 + + + +
EVMS - + - +
IB: TYMS
-35
Input
TYMS
GAPDH
-35
-35
B
MDA-MB-468
DeAcel - - + +
EVMS - + - +
TYMS -35
GAPDH -35
C
T47D
MDA-MB-468
Cross-link
MG132
EVMS
IB: TYMS
-70
Dimer
-35
Monomer
Low Exposure High Exposure Low Exposure High Exposure
Relative Protein Expression (Monomer/Dimer)
DMSO
EVMS
T47D
MDA-MB-468
D
T47D
MDA-MB-468
Relative mRNA levels
DMSO
RPMC
EVMS
OGA
OGT
E
T47D
MDA-MB-468
Thiamet-G - - + + - - + +
EVMS - + - + - + - +
TYMS
GAPDH
-35
-35
1.00 0.43 1.76 0.76
1.00 0.49 1.00 0.52
F
T47D
siNC
siOGT#1
siOGT#2
OGT -100
TYMS -35
GAPDH -35
Relative Protein Expression (to GAPDH)
OGT
TYMS
siNC siOGT#1 siOGT#2
G
Everolimus
mTOR
OGT
OGA
more stable
less stable
TYMS Dimer
Monomer
TYMS decreased stability with degron exposure
Ubiquitin-independent Proteolysis
Increased sensitivity to 5-Fluorouracil

fig. S5 Everolimus reduces O-GlcNAcylation and destabilizes thymidylate synthase via declined OGT level. (Related to Fig. 4-5)

(A) Acetylated TYMS was detected by co-immunoprecipitation using an anti-Ace-Lys antibody or an IgG control in MDA-MB-468 cells treated with MG132 (5 μM) with or without everolimus (100 nM) for 12 hours, followed by western blotting for TYMS. 2.5% input was used as a loading control.

(B) Western blot analysis of MDA-MB-468 cells treated with a 1X deacetylase inhibitor cocktail (400 nM Trichostatin A, 10 μM EX-527, 4 mM nicotinamide, and 2 mM sodium butyrate) with or without 10 nM everolimus (EVMS) for 24 hours.

(C) Cross-linking analysis of T47D and MDA-MB-468 cells treated with MG132 (5uM) and vehicle (DMSO) or 10nM everolimus (EVMS) for 12h, and proteins were cross-linked by disuccinimidyl suberate (DSS, 5mM) for 1h before cell lysis. Quantification of TYMS monomer /dimer equilibrium were shown as mean ± SEM in plots from dot-indicated biological replicates. Statistics were analyzed by multiple t-tests.

(D) qPCR analysis of *OGA* and *OGT* mRNA expressions were performed in T47D and MDA-MB-468 cells treated with vehicle (DMSO), 10 nM rapamycin (RPMC), or 10 nM everolimus (EVMS) for 48 hours. *ACTB* was used as the reference gene, and the relative gene expression was calculated using the $2^{(-\Delta\Delta Ct)}$ method. Data are presented as mean ± SEM from three independent experiments and were analyzed by two-way ANOVA.

(E) Western blot analysis of T47D and MDA-MB-468 cells treated with 10 nM everolimus (EVMS) and/or 2 μM Thiamet-G for 24 hours. The relative expression of TYMS was normalized to GAPDH, with the untreated group serving as the reference.

(F) T47D cells were transfected with either negative control siRNA (siNC) or OGT siRNA (siOGT #1, siOGT #2) for 48 hours. OGT and TYMS expression were then evaluated by Western blot. Quantification is shown as mean ± SEM from two independent experiments, analyzed by two-way ANOVA.

G) Schematic representation of the proposed mechanism by which everolimus enhances 5-fluorouracil efficacy. Everolimus downregulates OGT function, resulting in a less stable TYMS dimer. This destabilization promotes degron exposure, facilitating recognition by the proteasome degradation machinery. And the descend TYMS level increased vulnerability to 5-fluorouracil and capecitabine. $^{*}P < 0.05$; $^{**}P < 0.01$.

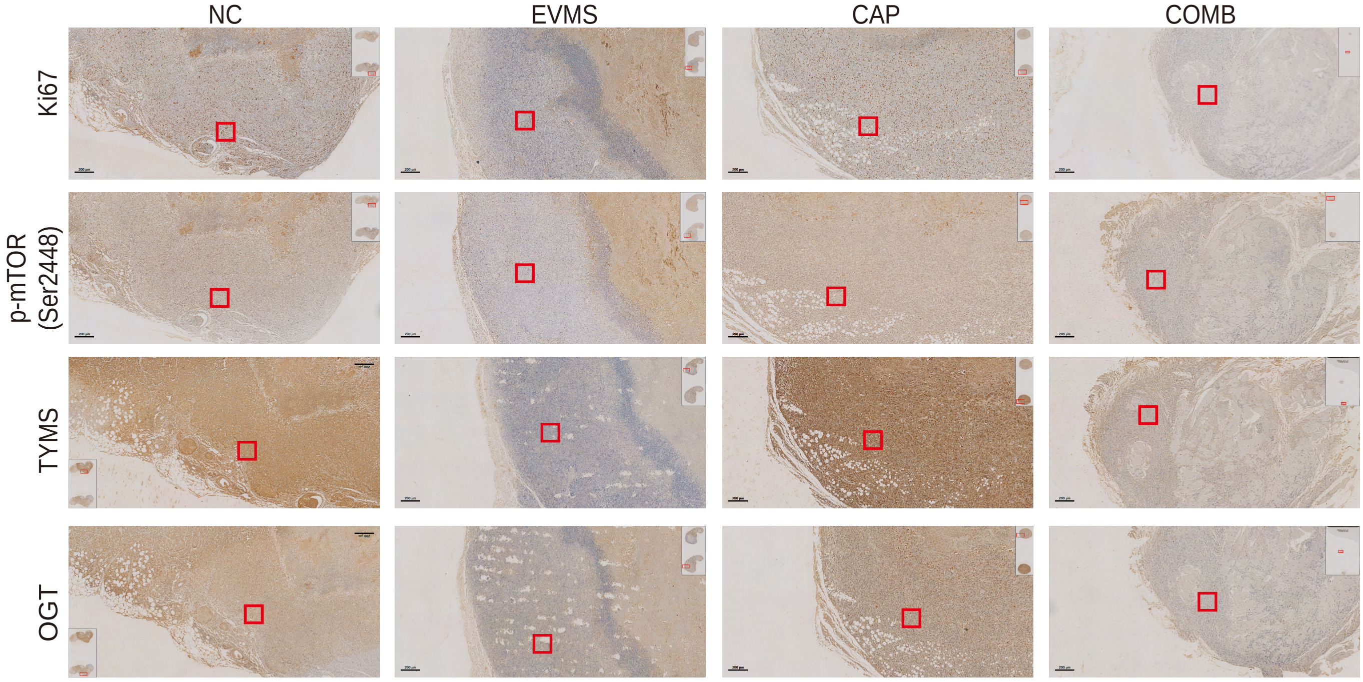

fig. S6 Low-magnification views of immunohistochemical (IHC) staining.

Representative whole-tissue section scans for Ki67, p-mTOR (S2448), TYMS, and OGT, corresponding to the higher-magnification images shown in Fig. 6F. For each scan, a red rectangle outlines the specific region that was displayed in Fig. 6F. A navigator view is provided as an inset. Scale bar is 200 μm.

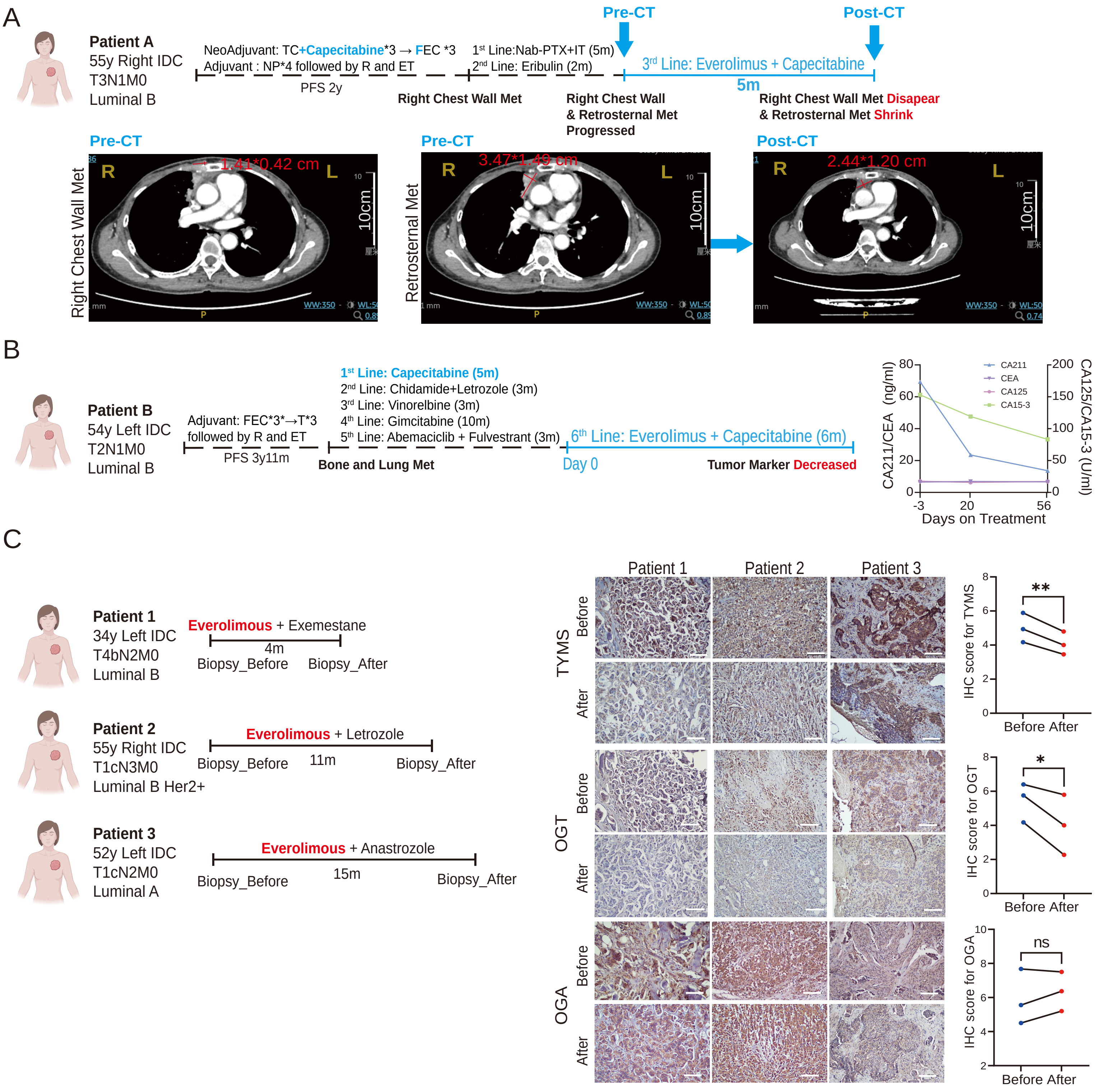

fig. S7 Everolimus combination therapy induces clinical responses in fluoropyrimidine-refractory breast cancer. **(A-B)** Case studies of two patients with metastatic, treatment-refractory Luminal B breast cancer who responded to everolimus combined with capecitabine. **(A)** Representative axial CT scans of Patient A and Patient B pre-treatment and after 4 or 5 months of combination therapy, demonstrating significant regression of target metastatic lesions (indicated by length and width, or arrows). **(B)** Line graphs depicting the kinetics of serum tumor marker levels (CEA, CA-211, CA-153, CA-125) over the course of treatment for Patients B. The days are synchronized to the start date of everolimus plus capecitabine treatment. Day 0 indicates the start of combination therapy.

(C) Tumor and treatment information for three paired everolimus-treated breast cancer samples of female patients from Sun Yat-Sen Memorial Hospital. Representative immunohistochemistry images of three paired breast cancer samples showing TYMS, OGT, and OGA staining before and after everolimus treatment. IHC scores for the three paired tumor specimens before and after everolimus treatment, are shown. Scale bars represent 100 μm. Paired t-tests were used for statistical analysis. Ns, not significant; *$P < 0.05$; **$P < 0.01$.
