# Supplementary material for: Everolimus destabilizes thymidylate synthase via suppressing its O-GlcNAcylation and sensitizes HER2-negative breast cancer to fluorouracil
Source: Cell Death Dis. 2026 Apr 4;17(1):456. doi: 10.1038/s41419-026-08715-z (PMC13184257; doi:10.1038/s41419-026-08715-z)
Supplement: Supplementary file 2 — Extended File 1 Uncropped WB image [file 41419_2026_8715_MOESM2_ESM.pdf]

Fig. 2H

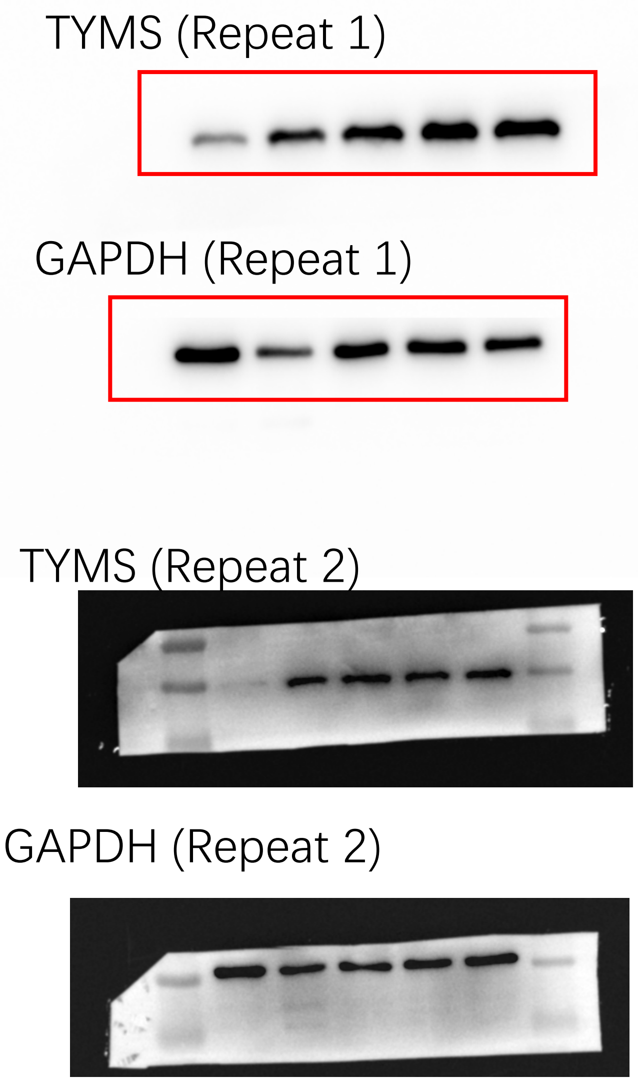

Fig. 3A-T47D

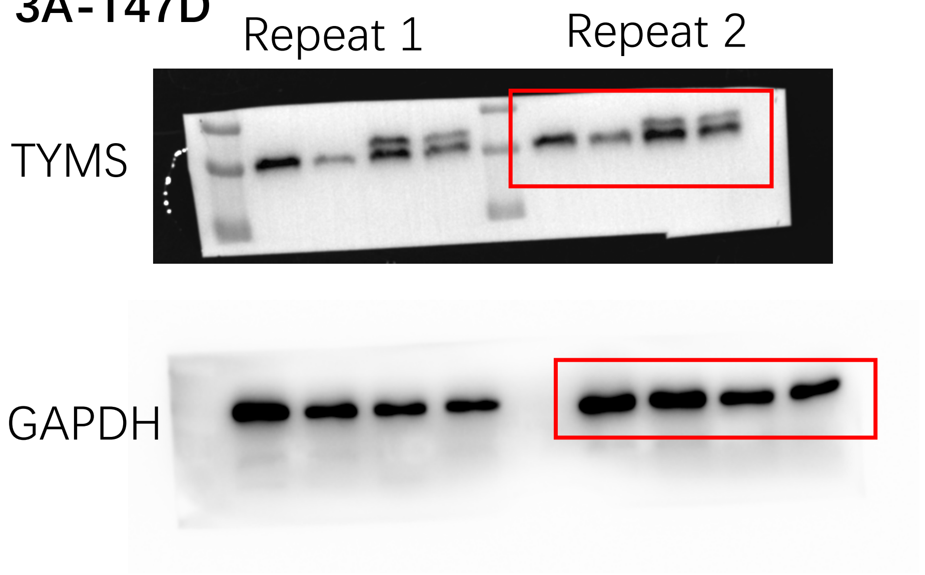

Fig. 3A-MDA-MB-468

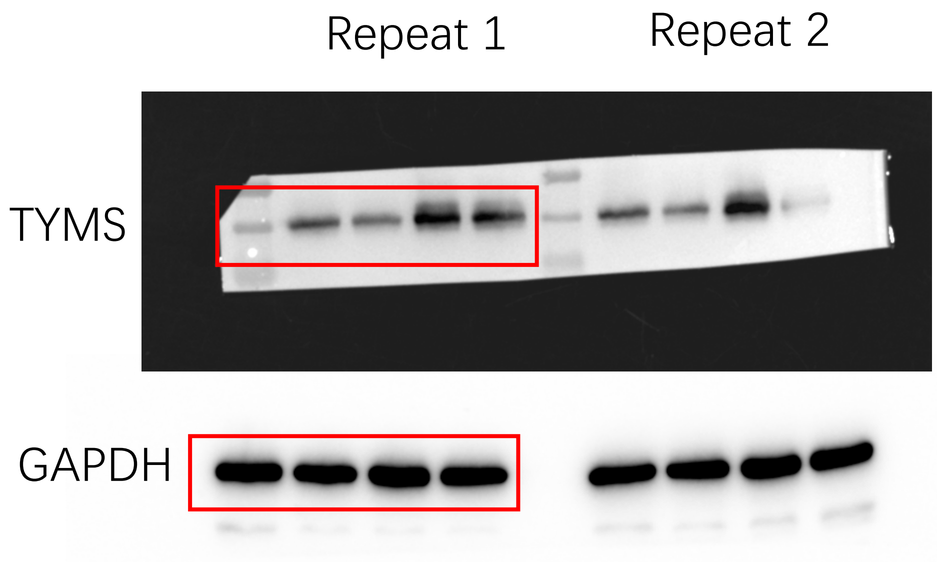

Fig. 3B

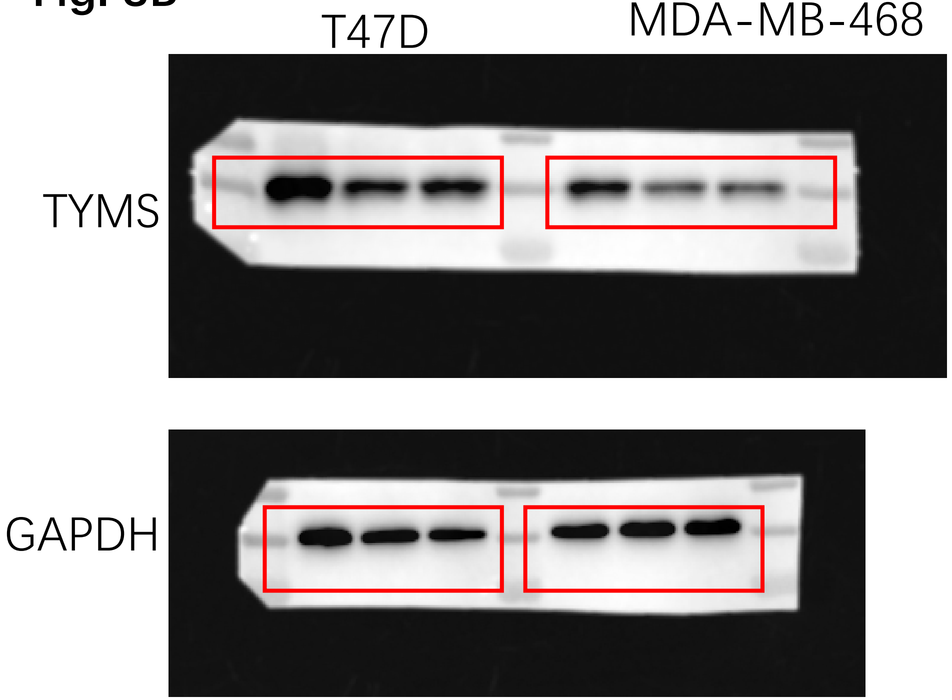

Fig. 3C-MDA-MB-468

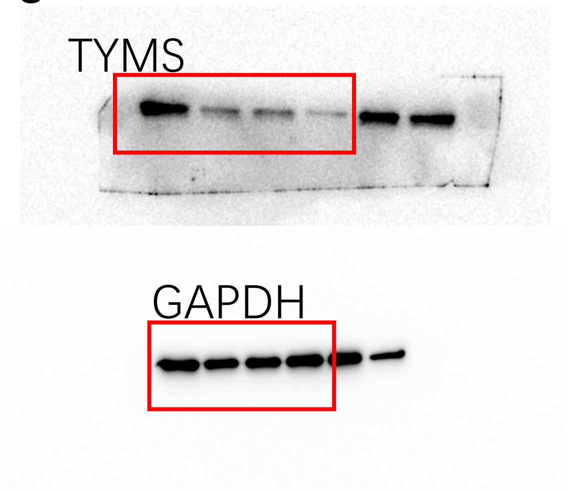

Fig. 3C-T47D

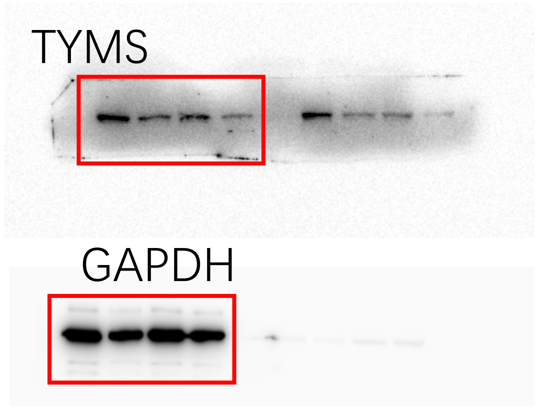

Fig. 3D

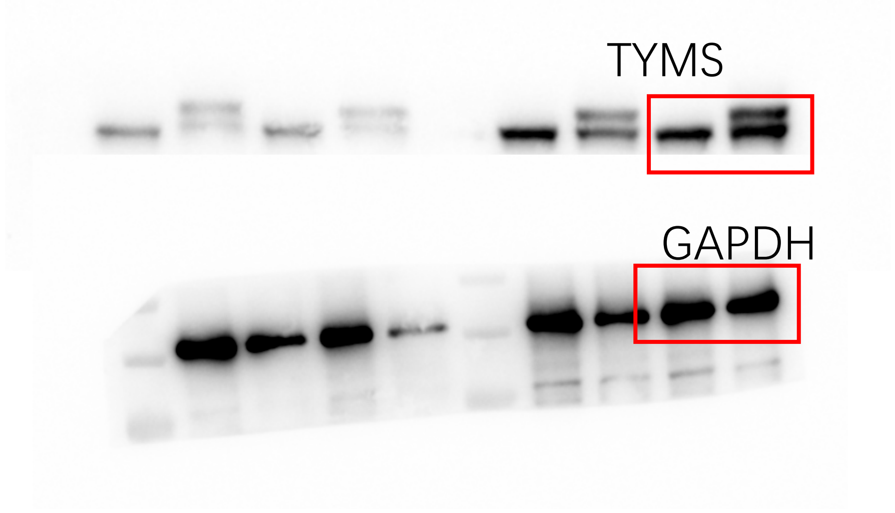

Fig. 4A

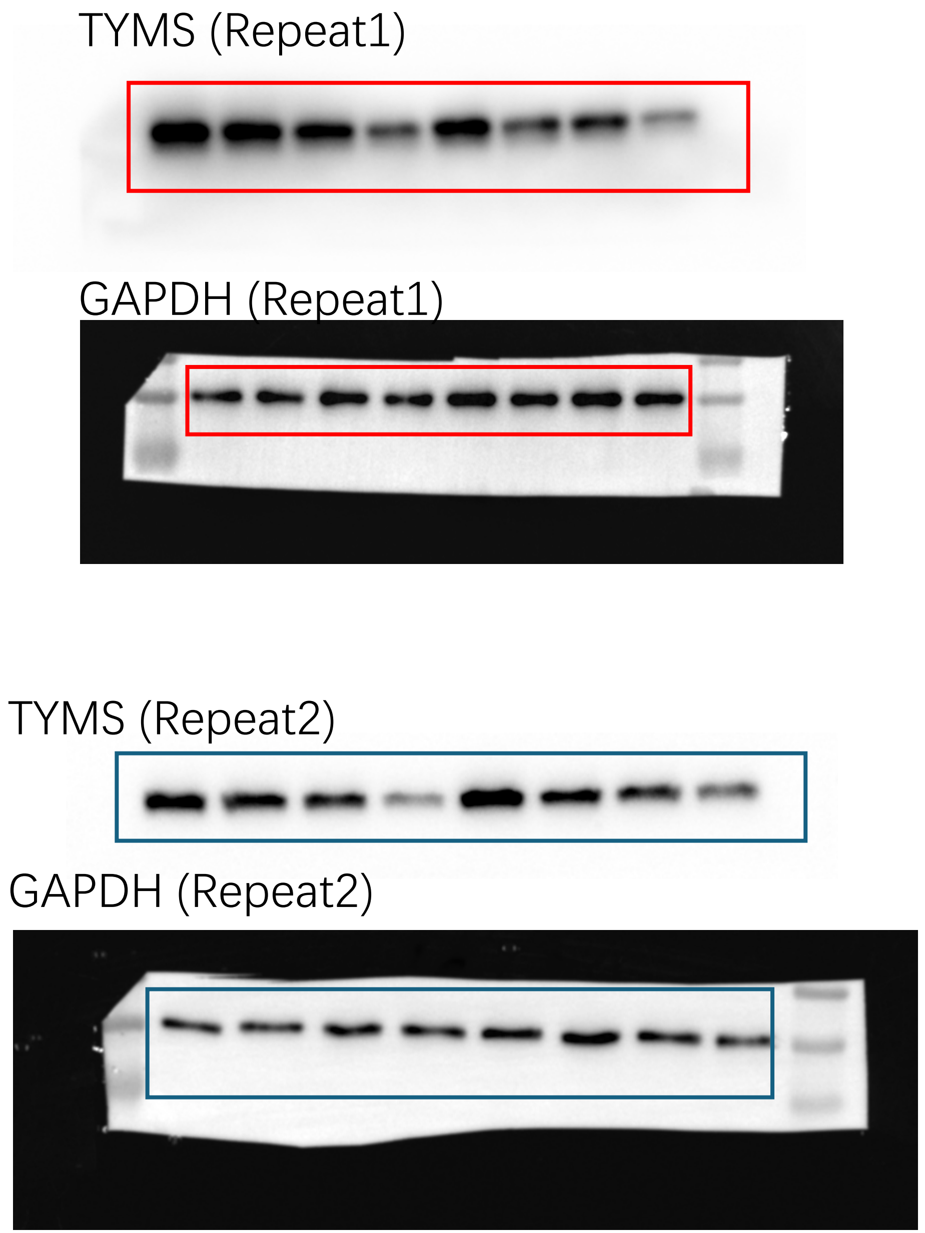
TYMS (Repeat1)
GAPDH (Repeat1)
TYMS (Repeat2)
GAPDH (Repeat2)

Fig. 4C

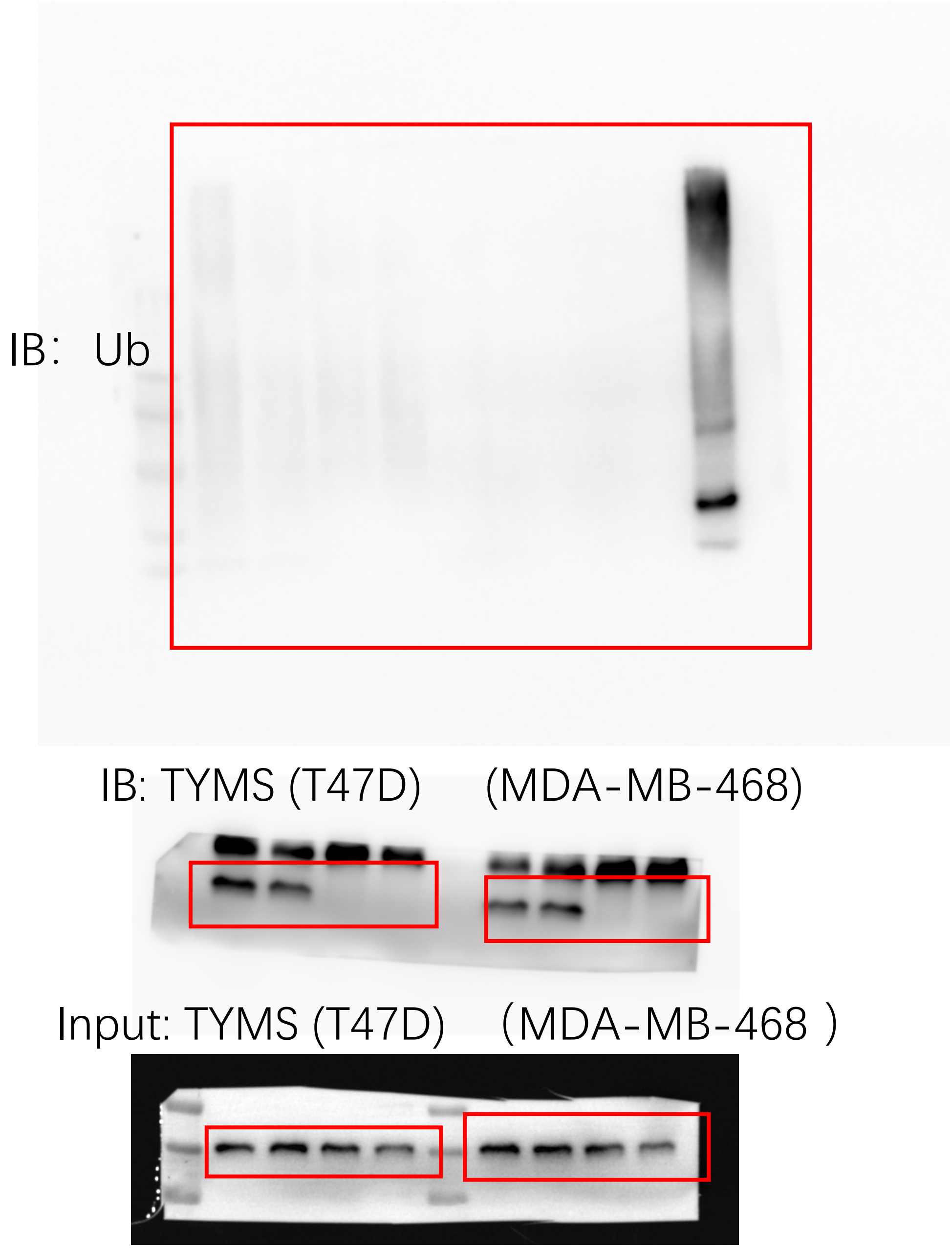
IB: Ub
IB: TYMS (T47D) (MDA-MB-468)
Input: TYMS (T47D) (MDA-MB-468)

Fig. 4B

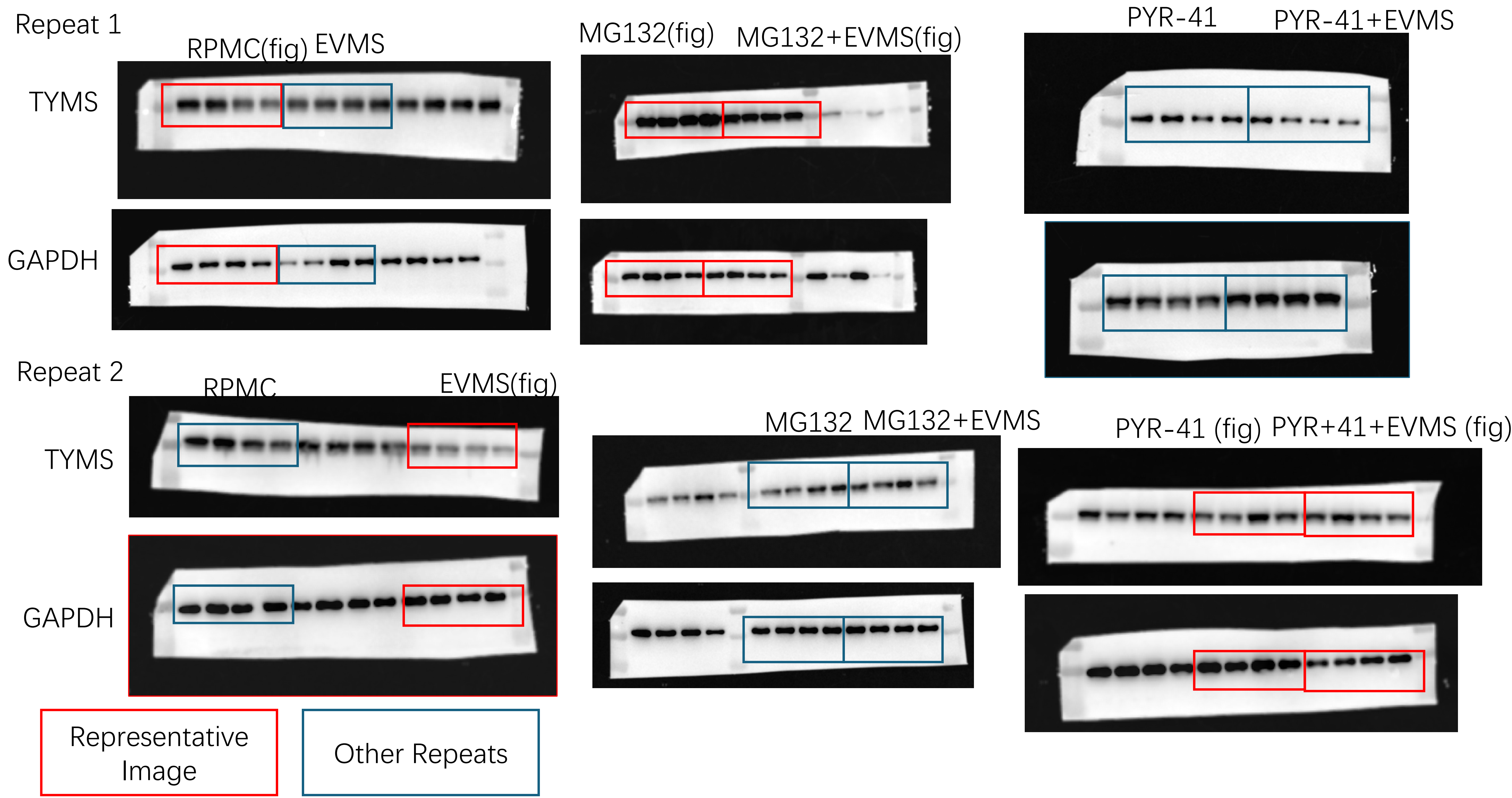

Repeat 1
RPMC(fig) EVMS
TYMS
GAPDH
MG132(fig) MG132+EVMS(fig)
PYR-41 PYR-41+EVMS
Repeat 2
RPMC EVMS(fig)
TYMS
GAPDH
MG132 MG132+EVMS
PYR-41 (fig) PYR+41+EVMS (fig)
Representative Image
Other Repeats

Fig. 4D

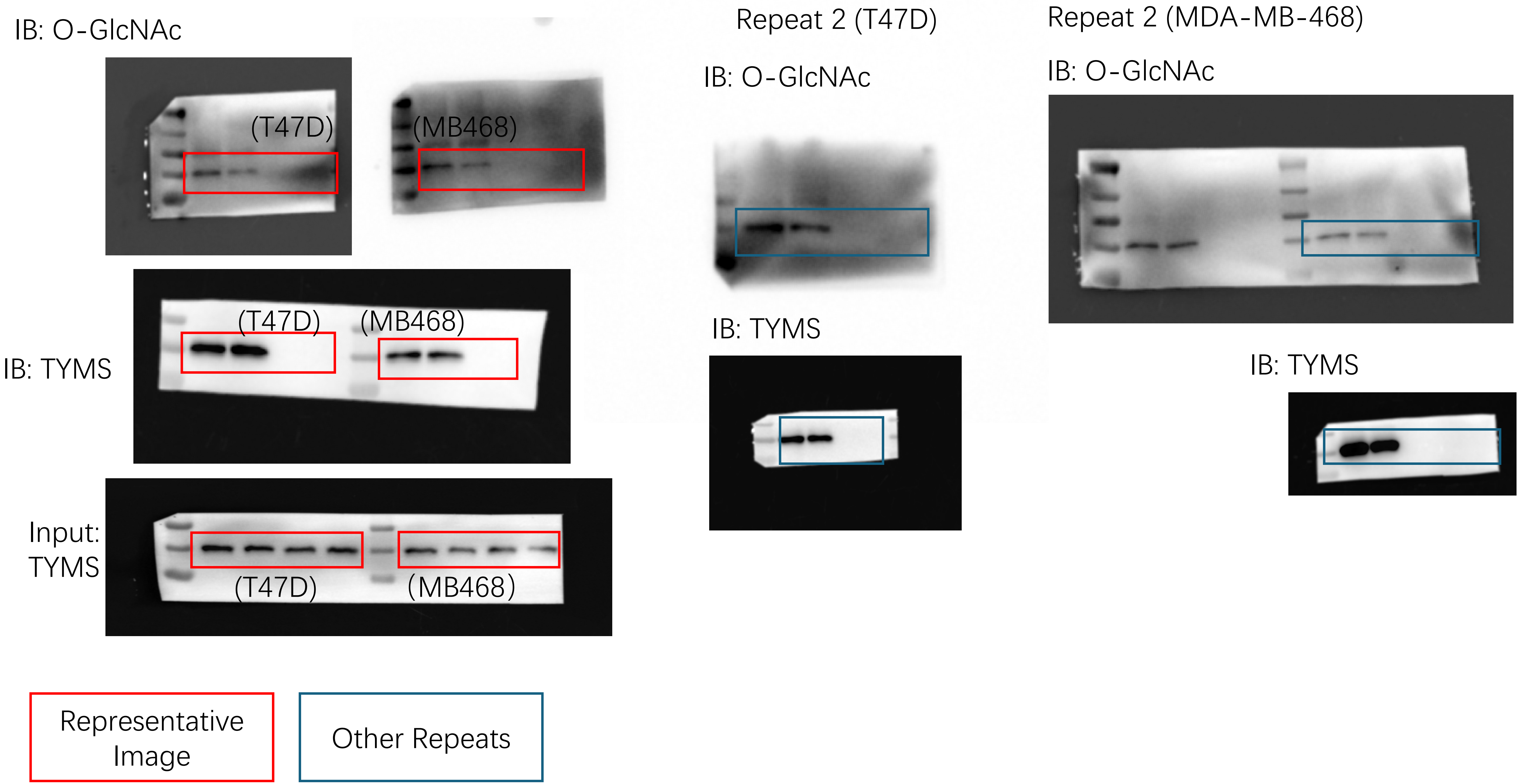
IB: O-GlcNAc
(T47D)
(MB468)
IB: TYMS
(T47D)
(MB468)
Input:
TYMS
(T47D)
(MB468)
Repeat 2 (T47D)
IB: O-GlcNAc
IB: TYMS
Repeat 2 (MDA-MB-468)
IB: O-GlcNAc
IB: TYMS
Representative Image
Other Repeats

Fig. 5A

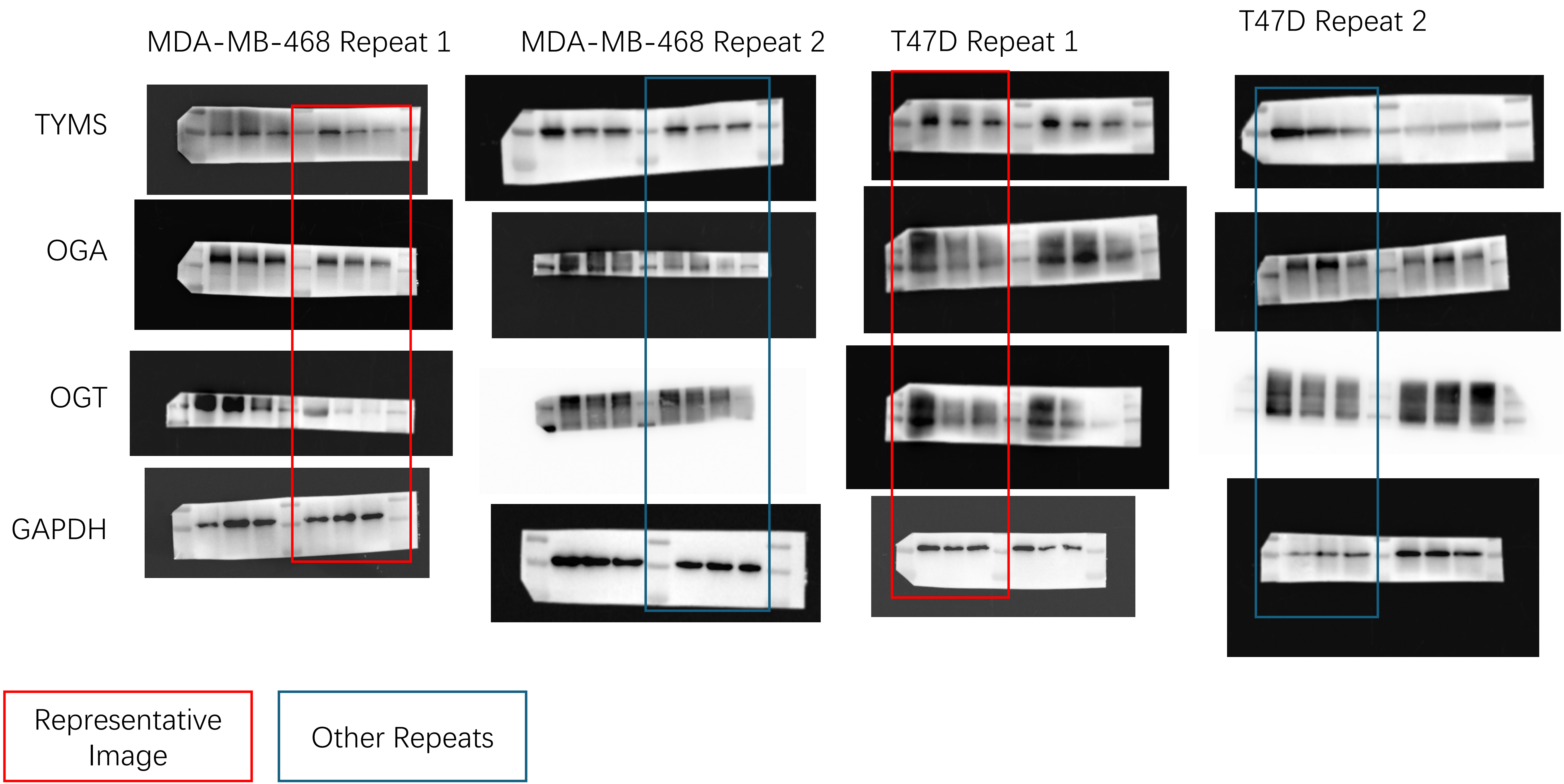
MDA-MB-468 Repeat 1
MDA-MB-468 Repeat 2
T47D Repeat 1
T47D Repeat 2
TYMS
OGA
OGT
GAPDH
Representative Image
Other Repeats

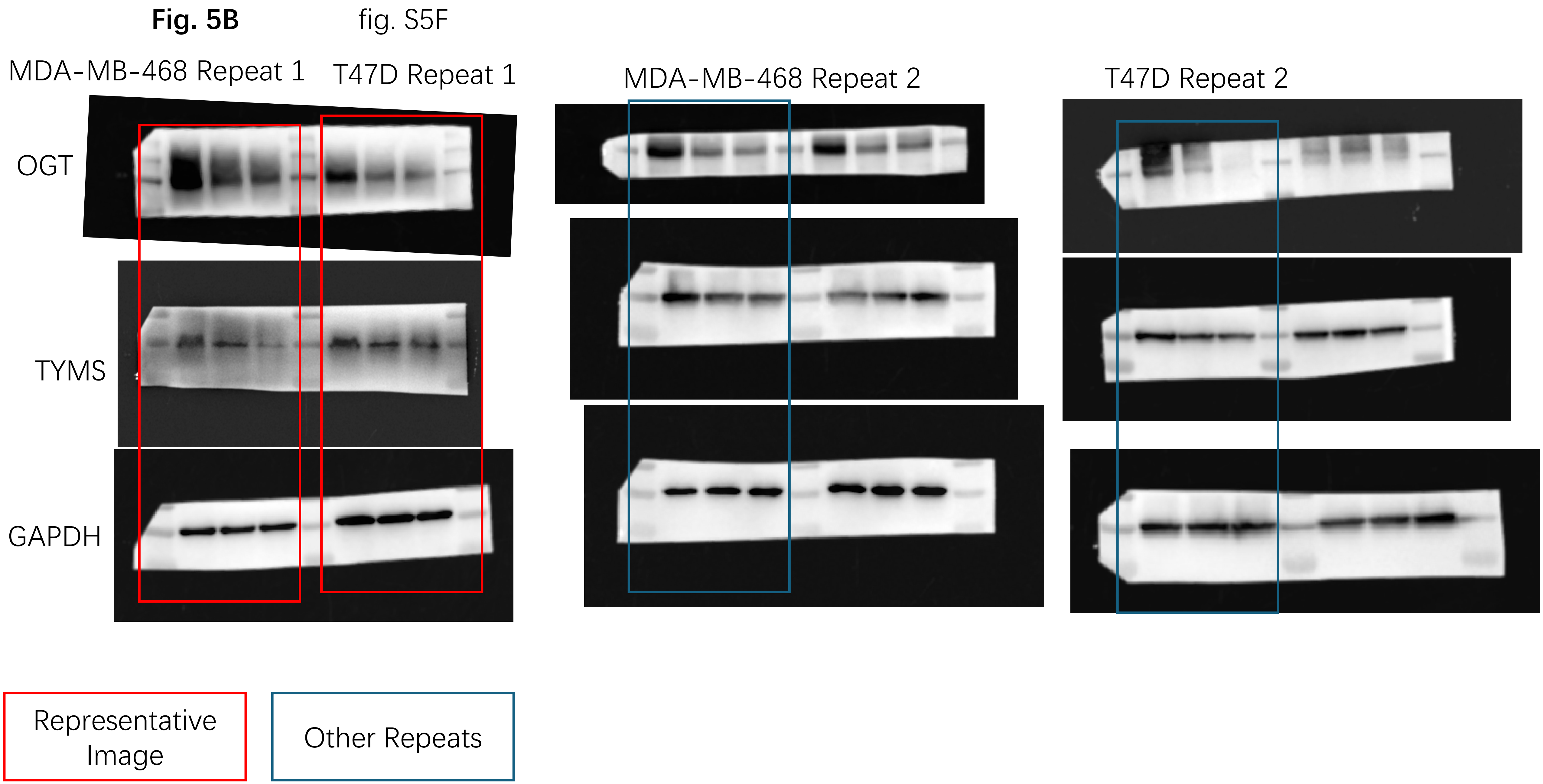

Fig. 5B
fig. S5F
MDA-MB-468 Repeat 1
T47D Repeat 1
MDA-MB-468 Repeat 2
T47D Repeat 2
OGT
TYMS
GAPDH
Representative Image
Other Repeats

Fig. 5C

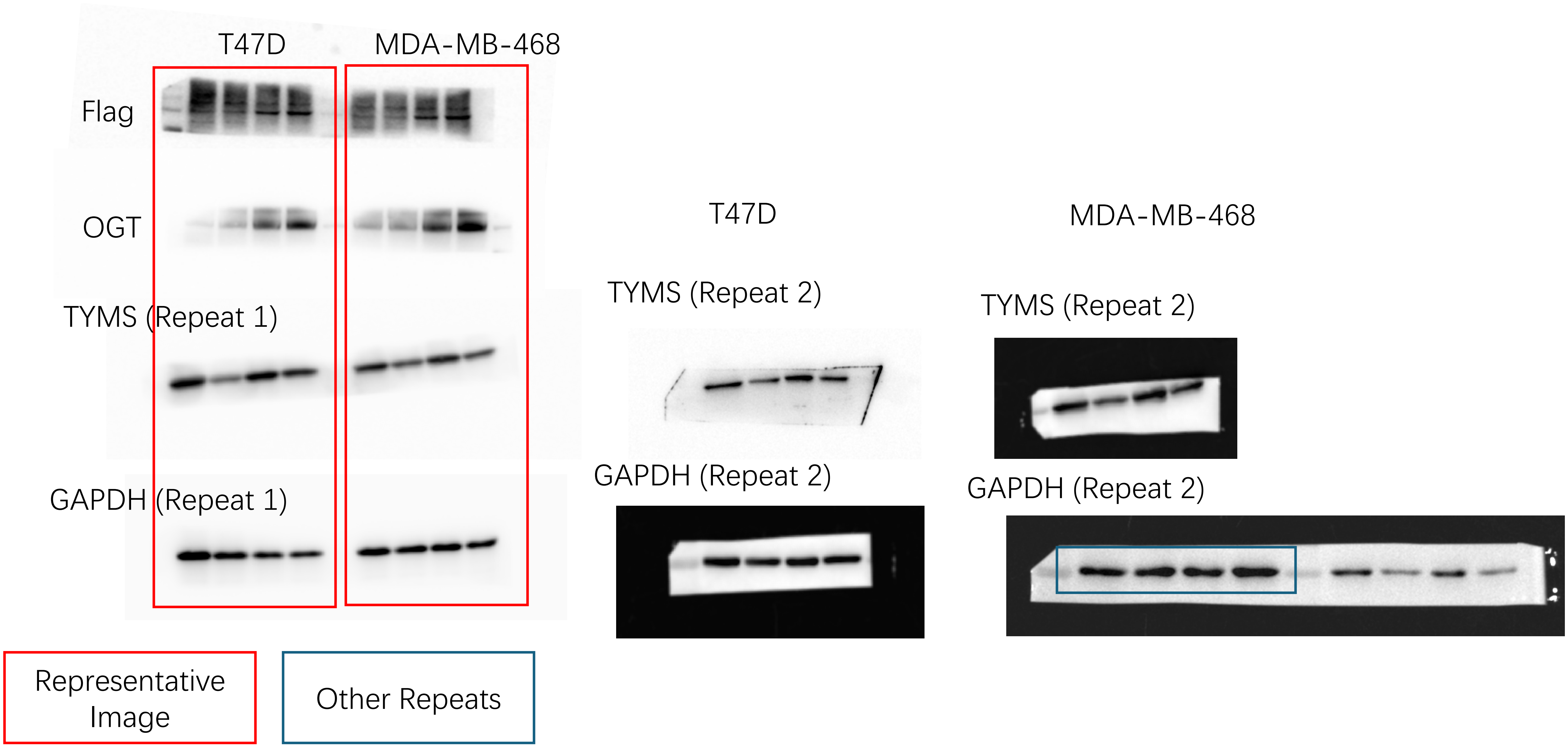
T47D
MDA-MB-468
Flag
OGT
TYMS (Repeat 1)
GAPDH (Repeat 1)
T47D
MDA-MB-468
TYMS (Repeat 2)
TYMS (Repeat 2)
GAPDH (Repeat 2)
GAPDH (Repeat 2)
Representative Image
Other Repeats

fig. S4A

TYMS (T47D)

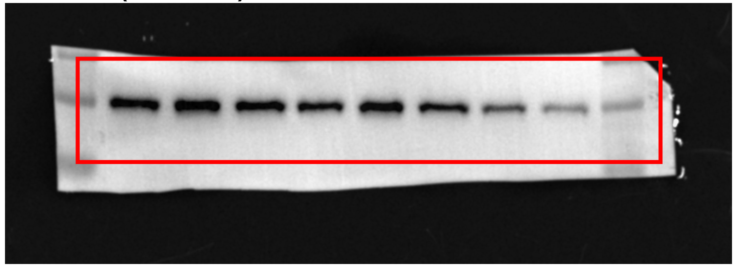

GAPDH (T47D)

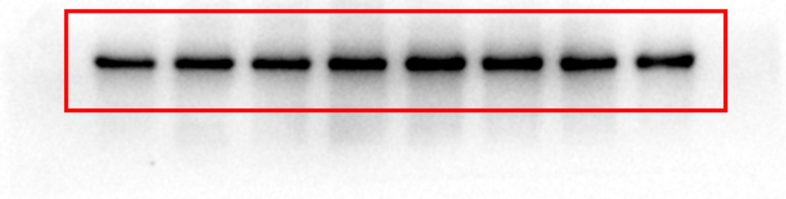

fig. S4B

TYMS (T47D)

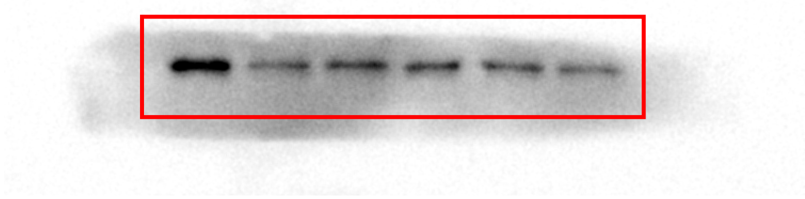

GAPDH (T47D)

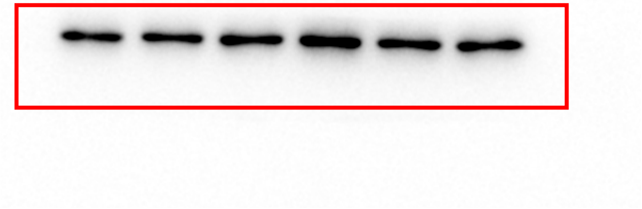

fig. S4A

TYMS (MDA-MB-468)

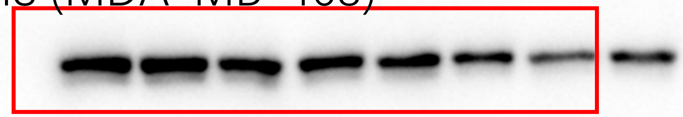

GAPDH (MDA-MB-468)

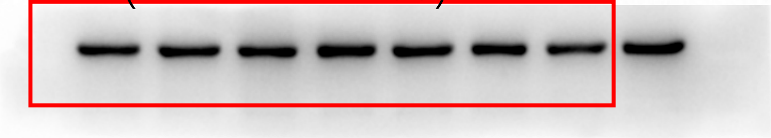

fig. S4B

TYMS (MDA-MB-468)

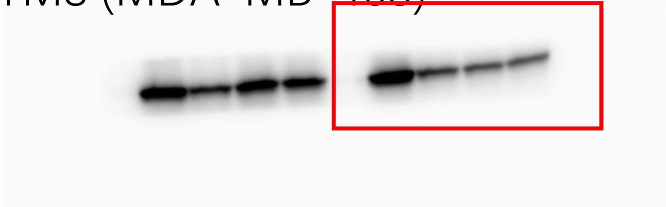

GAPDH (MDA-MB-468)

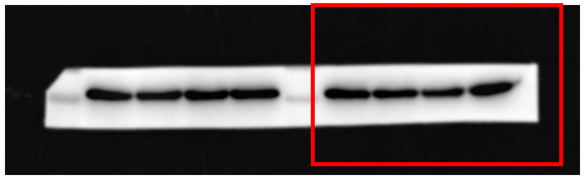

fig. S4D

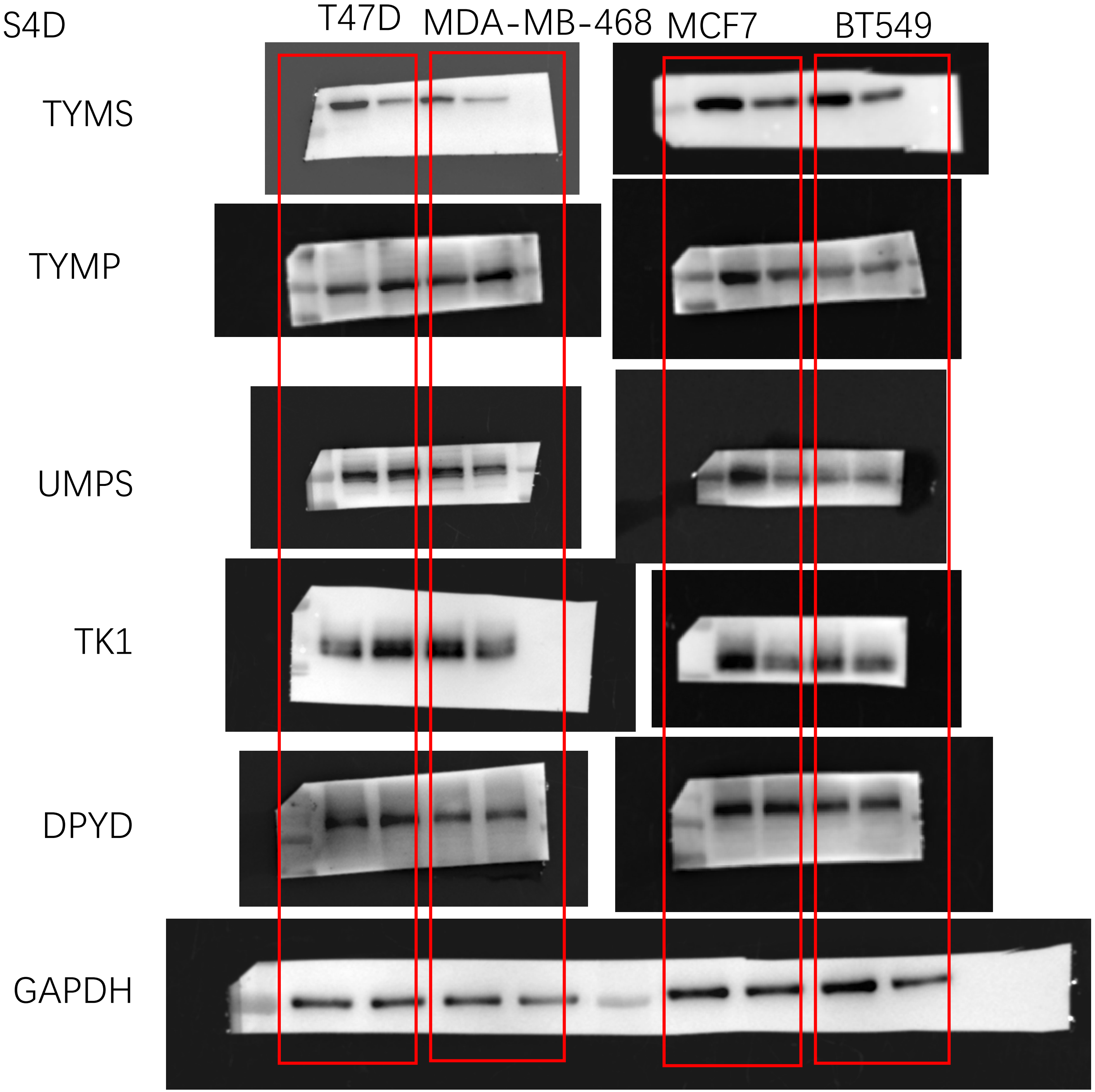

fig. S4F

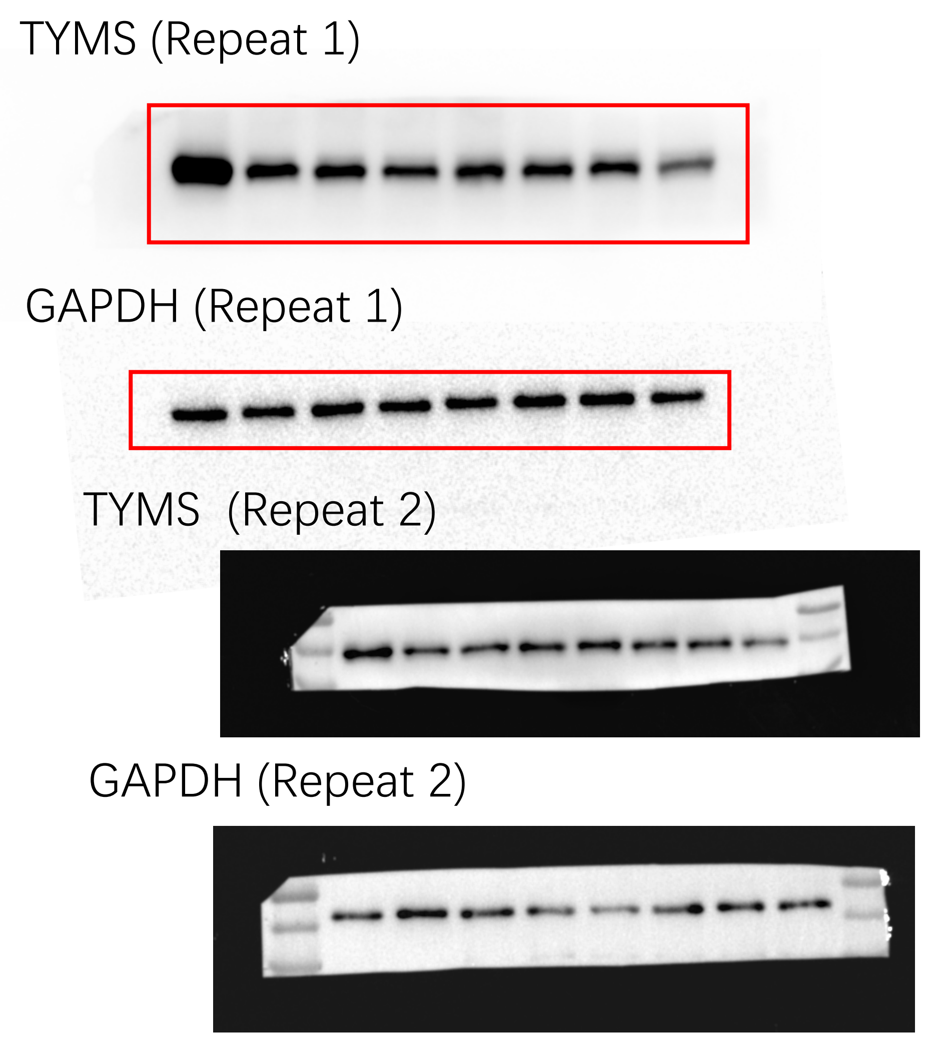

fig. S4G

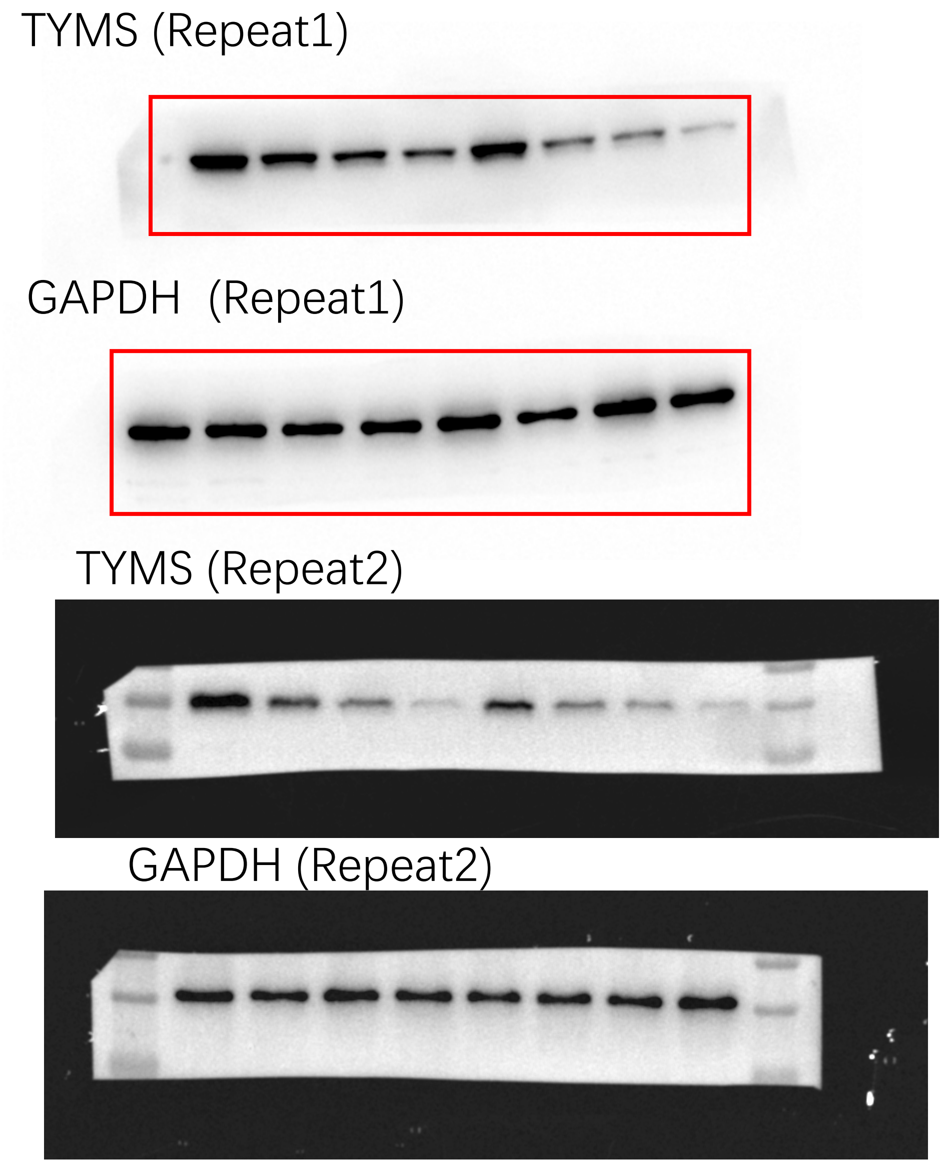

fig. S4H

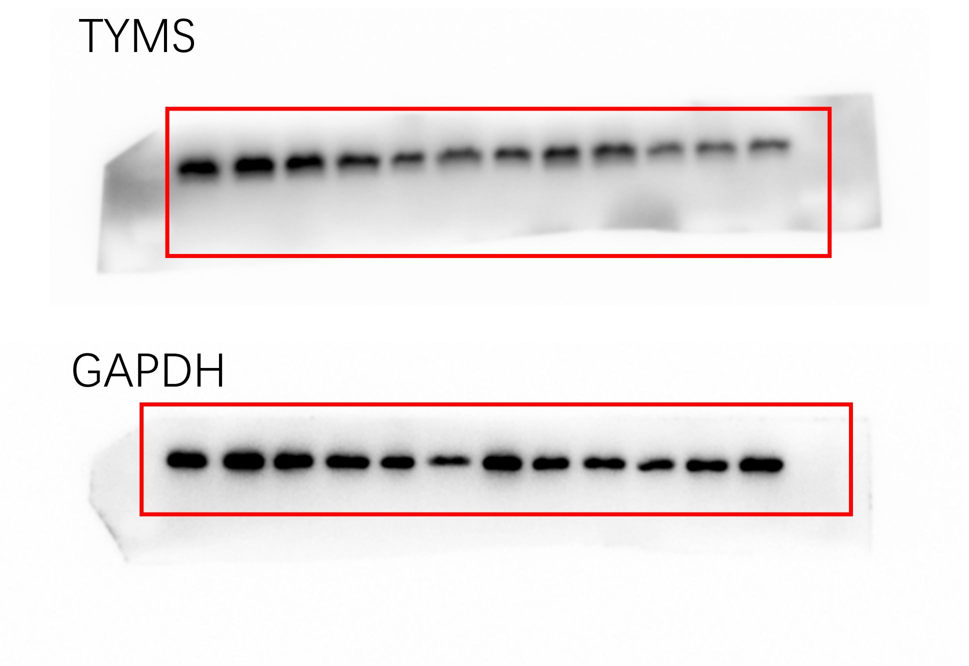

fig. S4I

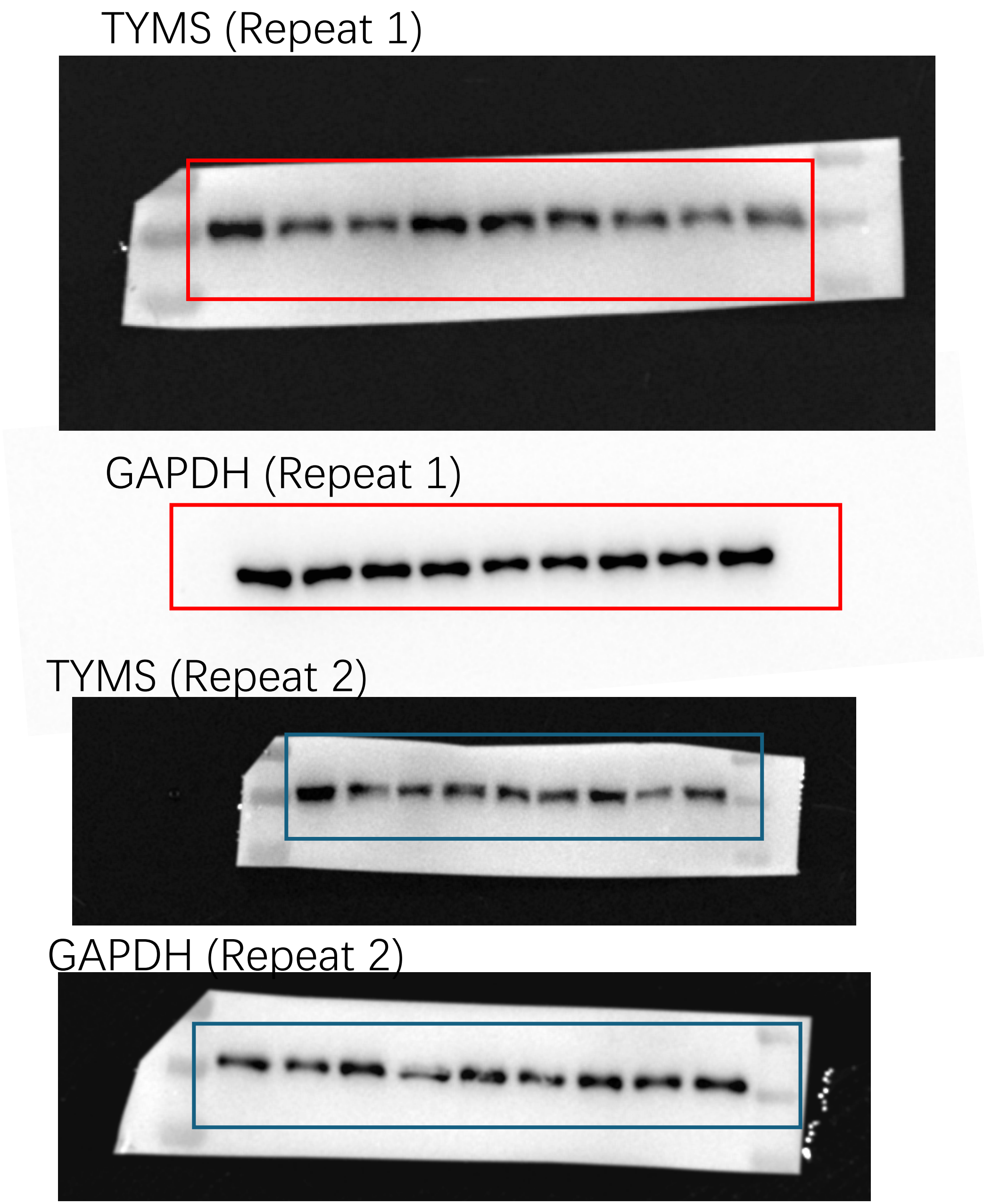

fig. S5A

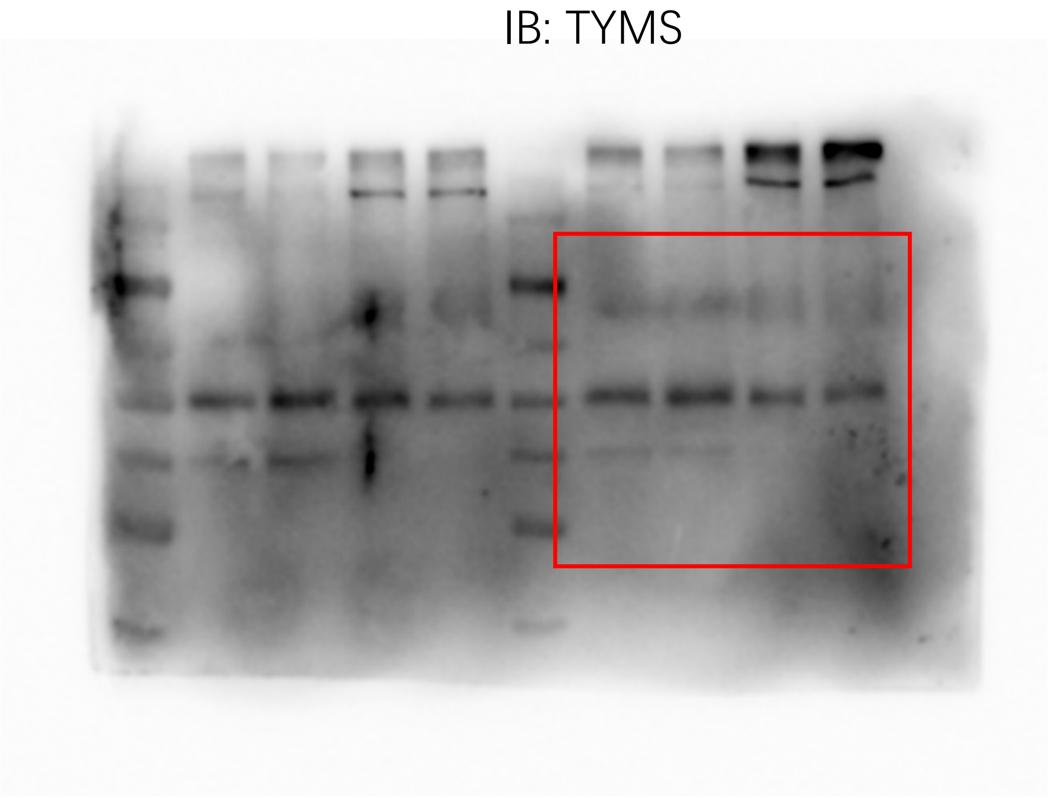

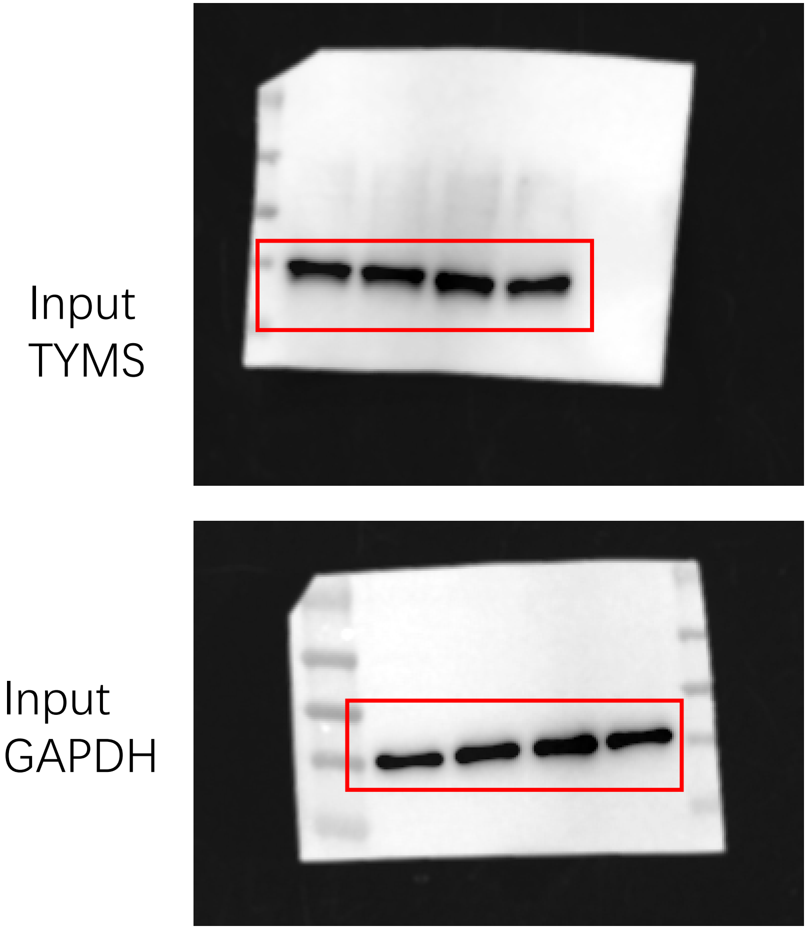

fig. S5B

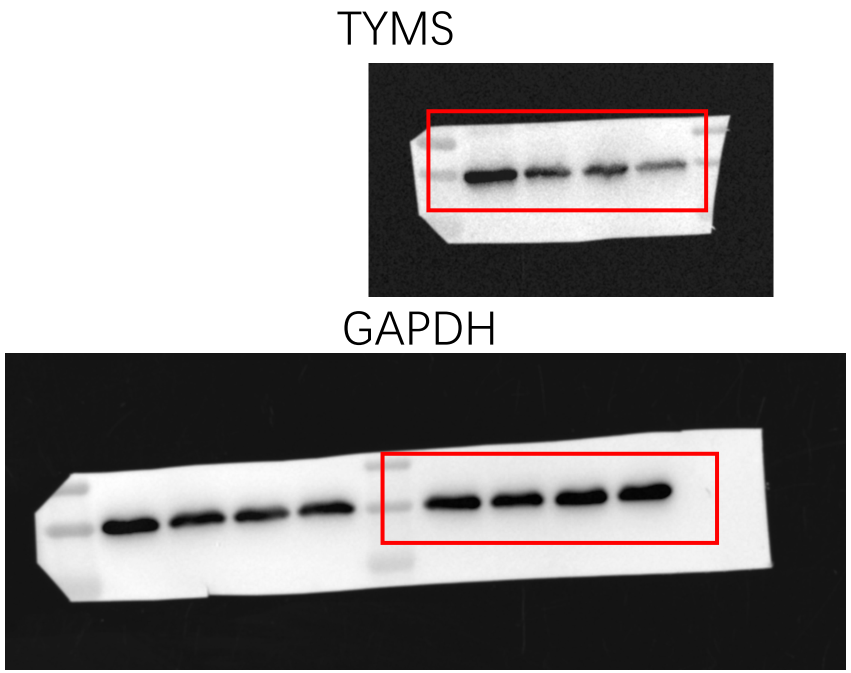

fig. S5E

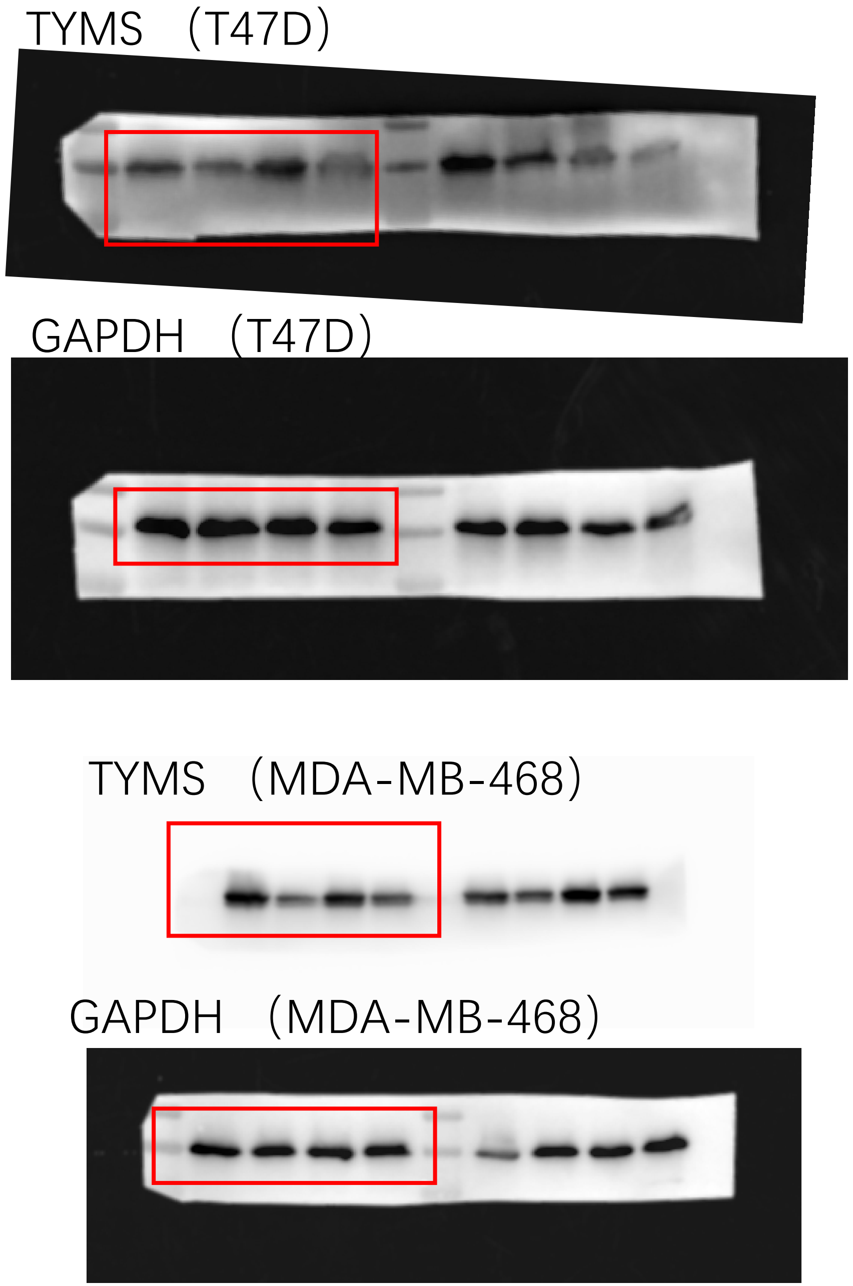

fig. S5C

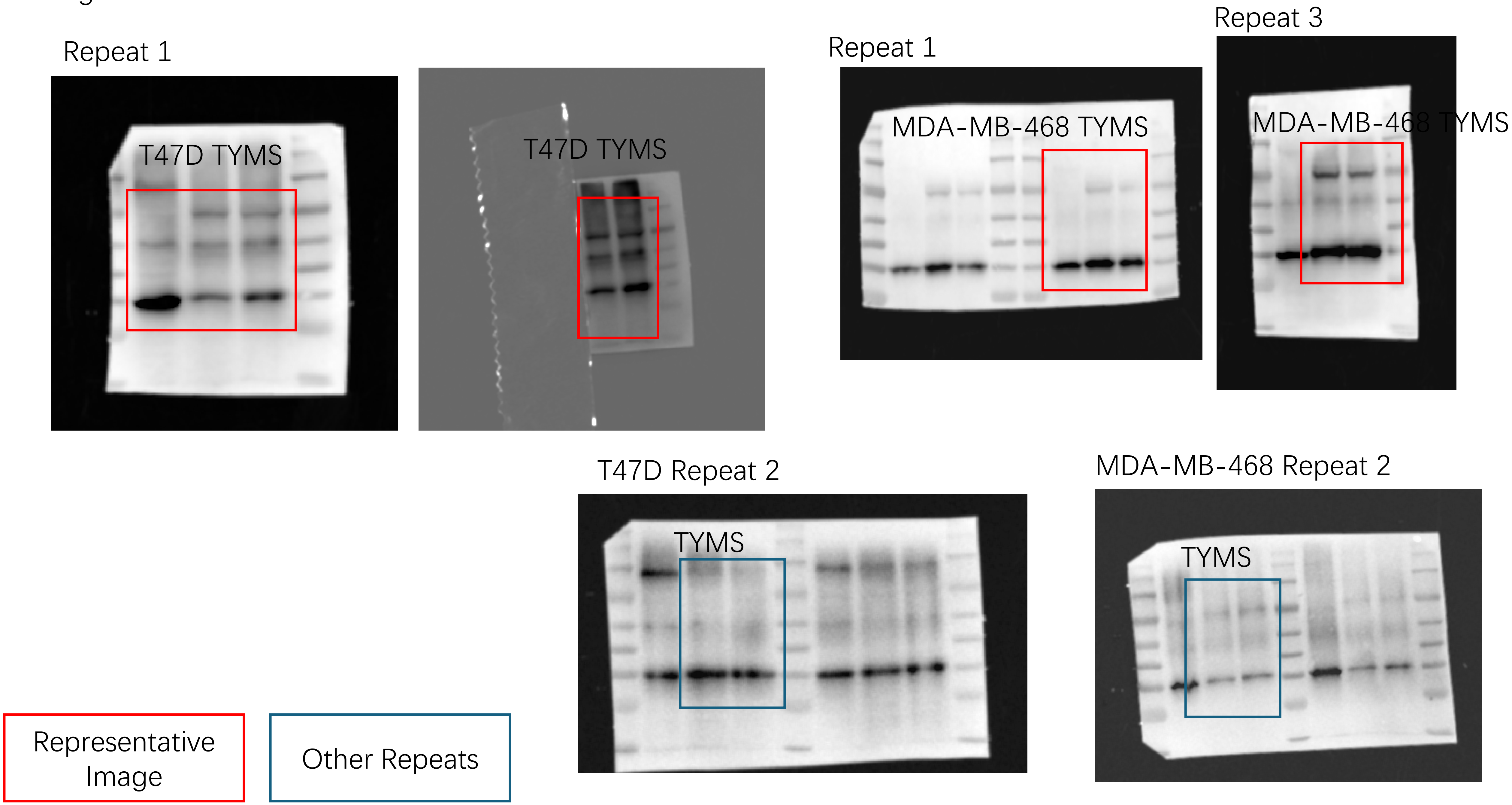
Repeat 1
T47D TYMS
T47D TYMS
Repeat 1
MDA-MB-468 TYMS
Repeat 3
MDA-MB-468 TYMS
T47D Repeat 2
TYMS
MDA-MB-468 Repeat 2
TYMS
Representative Image
Other Repeats
